# To Vaccinate or Not to Vaccinate—Reasons of Willingness and Reluctance of Students against SARS-CoV-2 Vaccination—An International Experience

**DOI:** 10.3390/ijerph192114012

**Published:** 2022-10-27

**Authors:** Ryszard Sitarz, Alicja Forma, Kaja Karakuła, Dariusz Juchnowicz, Jacek Baj, Jacek Bogucki, Joanna Rog, Michael L. Tee, Cherica A. Tee, Josefina T. Ly-Uson, Md. Saiful Islam, Md. Tajuddin Sikder, Ahmed Hashem El-Monshed, Ahmed Loutfy, Muhammad Fazal Hussain Qureshi, Munib Abbas, Shafaq Taseen, Mahira Lakhani, Cuiyan Wang, Xiaoyang Wan, Yilin Tan, Riyu Pan, Roger Ho, Saikarthik Jayakumar, Saraswathi Ilango, Senthil Kumar, Ángel A. Ruiz-Chow, Adriana Iturbide, David D. González-Mille, Linh Phuong Doan, Hanna Karakuła-Juchnowicz

**Affiliations:** 1 I Department of Psychiatry, Psychotherapy and Early Intervention, Medical University of Lublin, 20-059 Lublin, Poland; 2 Department of Forensic Medicine, Medical University of Lublin, 20-059 Lublin, Poland; 3 Department of Psychiatry and Psychiatric Nursing, Medical University of Lublin, 20-059 Lublin, Poland; 4 Department of Anatomy, Medical University of Lublin, 20-059 Lublin, Poland; 5 Department of Organic Chemistry, Faculty of Pharmacy, Medical University of Lublin, 20-059 Lublin, Poland; 6 College of Medicine, University of the Philippines Manila, Manila 1000, Philippines; 7 Department of Public Health and Informatics, Jahangirnagar University, Savar, Dhaka 1342, Bangladesh; 8 Centre for Advanced Research Excellence in Public Health, Savar, Dhaka 1342, Bangladesh; 9 Department of Psychiatric and Mental Health Nursing, Faculty of Nursing, Mansoura University, Mansoura 35511, Egypt; 10 Nursing Department, College of Health and Sport Sciences, University of Bahrain, Manama 32038, Bahrain; 11 Department of Pediatric Nursing, Faculty of Nursing, Beni-Suef University, Beni-Suef 62521, Egypt; 12 Medical College, Ziauddin Medical University, Saharah-e-Ghalib, Clifton, Karachi 75600, Pakistan; 13 Department of Medicine, Karachi Medical and Dental College, North Nazimabad, Karachi 74700, Pakistan; 14 Faculty of Education, Huaibei Normal University, Huaibei 235000, China; 15 Anqing Normal University, Anqing 246133, China; 16 Department of Psychological Medicine, National University of Singapore, Singapore 117599, Singapore; 17 Department of Basic Medical Science and Department of Medical Education, College of Dentistry, Al Zulfi, Majmaah University, Al Majmaah 11952, Saudi Arabia; 18 Madha Medical College and Research Institute, Kovoor, Chennai 600128, India; 19 Centro Médico ABC, Mexico City 05348, Mexico; 20 Duy Tan University, 254 Nguyen Van Linh, Danang 550000, Vietnam

**Keywords:** SARS-CoV-2, COVID-19, COVID-19 vaccine, pandemic, students, psychological and psychiatric support, supplements, influenza

## Abstract

Despite the vaccine against the severe acute respiratory syndrome coronavirus 2 (SARS-CoV-2) being reported to be safe and effective, the unwillingness to vaccinate and doubts are still common. The aim of this international study was to assess the major reasons for the unwillingness to vaccinate in a group of students from Poland (n = 1202), Bangladesh (n = 1586), India (n = 484), Mexico (n = 234), Egypt (n = 566), Philippines (n = 2076), Pakistan (n = 506), Vietnam (n = 98) and China (n = 503). We conducted an online cross-sectional study that aimed to assess (1) the percentage of vaccinated and unvaccinated students and (2) the reasons associated with willingness/unwillingness to the vaccine. The study included 7255 respondents from 9 countries with a mean age of 21.85 ± 3.66 years. Only 22.11% (n = 1604) of students were vaccinated. However, the majority (69.25%, n = 5025) expressed a willingness to be vaccinated. More willing to vaccinate were students in informal relationships who worked mentally, used psychological/psychiatric services before the pandemic, and studied medicine. There are cultural differences regarding the reasons associated with the unwillingness to vaccinate, but some ‘universal’ might be distinguished that apply to the whole group.

## 1. Introduction

Before the widespread use of vaccines in the 20th century, infectious diseases took their toll worldwide, leading to high rates of morbidity and death. In modern years, vaccines contributed to the eradication of smallpox and elimination of poliomyelitis, as well as to the control of measles, rubella, tetanus, diphtheria, and Haemophilus influenzae type b [1]. Despite such achievements, in the 21st century, people began to doubt the value of vaccines. The so-called ‘Crisis of public confidence’ is a phenomenon that is increasingly discussed by experts [2].

Nowadays, reluctance to vaccinate is alarmingly common regardless of age, characteristics of the population, culture, or religion [3,4,5]. Such attitudes can be found in the progressive technological development, which made social media allow immediate access to information that can be shared on a large scale without editorial supervision [6]. Misinformation is conducive to conspiracy theories and could shape public distrust towards immunization [7]. What is more, concerns are raised due to the adverse symptoms of the coronavirus disease 2019 (COVID-19) vaccine, including those related to the nervous system, acute myocarditis, pericarditis, purpuric rash, thrombocytopenia, vein thrombosis, chest pain, developing the new abnormal electrocardiographic record, eosinophilic panniculitis, and other dermatologic conditions [8,9,10,11,12,13,14,15,16,17]. Additionally, the discussion is incited by the issues of the population with chronic diseases that hesitate to vaccinate [18,19,20,21]. 

Going one step further, the concept of “infodemic” cannot be ignored in the context of the prevailing uncertainty about COVID-19 vaccination [22,23,24]. The decision to vaccinate seems to be driven not only by scientific and economic but also by psychological, political, social, and cultural factors. Public trust in vaccines varies and depends on personal experiences, knowledge, beliefs, religious or political affiliation, and socioeconomic status [25]. The pandemic shows cultural differences in human behavior, giving research space to transcultural psychiatry. Some of the studies indicate that there is a relationship between the willingness to vaccinate and socioeconomic status, past SARS-CoV-2 infection, or education level in a population of students specifically [26,27,28]. Anyway, the close attention to the latest news about the SARS-CoV-2 vaccine, fear of SARS-CoV-2 infection and the fear of relatives being infected, general levels of stress and fear as well as the fear of possible side effects after vaccination are also reported to influence students’ decision about the vaccination [29,30].

Even if the elderly is more exposed to the lethal effects of COVID-19 infection, young adults are also sensitive to the disease and more likely to spread the virus dynamically [31,32]. Unfortunately, young people perceive themselves as not at risk of contracting COVID-19, which has led to reports of their poor adherence to safety compliance [33]. In the case of asymptomatic transmission, the virus is spreading unknowingly, and vaccination among young adults seems to be crucial, especially as there have been more cases of severe symptoms among the youth population [34]. Many countries around the world introduced preventive measures during the pandemic, which also had to be subjected to students in their daily academic needs, high mobility, and intense social activities, and who, like no other group, are so representative when it comes to finding themselves at decisive moments for their future life [35]. Gaining opinions among university students should be highly valued as they are the young generation shaping the vision of a forthcoming society. Especially the position of medical students may represent the opinion of the future healthcare workers’ environment, which may contribute to the success of preventive pro-health actions in modern society [36,37].

When designing the study, we wanted to hear the voice of young, open-minded, and educated people studying various fields of study, coming from different parts of the world, with different socioeconomic statuses, and to find out beyond cultural, political, and religious divisions what kind of universal uncertainties are behind the delay to vaccinate against COVID-19, especially when the virus has become global in scope.

This cross-sectional study aims to—(1) investigate the percentage of students vaccinated against SARS-CoV-2 from nine countries (i.e., Poland, Bangladesh, India, Mexico, Egypt, Philippines, Pakistan, Vietnam, and China), (2) assess the factors associated with the willingness to vaccinate, and (3) evaluate the reasons for the reluctance to vaccinate.

## 2. Materials and Methods

### 2.1. Study Design and Data Collection

Our research group prepared an online questionnaire that aimed to be distributed among universities in nine countries (i.e., Poland, Bangladesh, India, Mexico, Egypt, Philippines, Pakistan, Vietnam, and China). Before survey preparation, we independently performed a literature review regarding the impact of the COVID-19 pandemic on the student’s mental health along with their attitudes towards the COVID-19 vaccine. The questionnaire was composed of several parts, including questions related to (1) socio-demographic information, (2) economic situation, (3) health-related factors, and (4) attitudes toward COVID-19 vaccination. An anonymous online cross-sectional questionnaire was distributed on the 12th of April 2021 via social media (e.g., Facebook, Twitter, Instagram, etc.) and via e-mails to Polish students from medical, social, technical, artistic, humanistic, and science universities. Further, the questionnaire was distributed to universities in other countries, including Bangladesh, India, Mexico, Egypt, Philippines, Pakistan, Vietnam, and China. The survey could be completed by each individual at an estimated time of 35–40 min. Before completing the questionnaire, the respondents were asked to provide consent to perform the survey freely. All of the answers given by the respondents were confidential, and only the researchers had the possibility to access the answers. Only statistically significant results were described and analyzed in more detail. The language used for the survey instrument was elaborated for individual countries. The researchers from the collaborating countries (Bangladesh, India, Mexico, Egypt, Philippines, Pakistan, Vietnam, and China) were chosen and agreed to take part in this study based on their previous experience and research on the impact of the COVID-19 pandemic on the students specifically. While searching for the collaborating countries, we primarily focused on whether other researchers had already published papers devoted to this matter. While sending research collaboration proposals, ultimately, we gathered positive responses from all of the aforementioned countries. 

### 2.2. Measures

The questionnaire was composed in a way to provide the most crucial information about the respondents: (A) The sociodemographic information included questions regarding (1) gender, (2) age, (3) place of residence, (4) marital status, (5) having children, (6) housing situation, (7) field of study, and (8) mode of conducting classes—online, hybrid, or stationary; (B) Economic situation including working status; (C) Health-related factors including (1) usage of psychological/psychiatric services before and during a pandemic, (2) supplement intake before and during the pandemic, and (3) being vaccinated against influenza; and (D) Attitudes toward COVID-19 vaccination.

## 3. Statistical Analysis

In the statistical calculations, the analysis of the number of responses in groups was used, the percentage distribution of responses, along with the Chi-square statistics. All calculations were made with Statistica v.13 program; Statistica software—Polish version from StatSoft Corporation Poland, the partner of Tibco Corporation, Palo Alto, California, USA. In the statistical analysis, the critical level of statistical significance was assumed to be less than 0.05.

## 4. The Pandemic Situation during the Questionnaire Distribution

**Figure 1:**
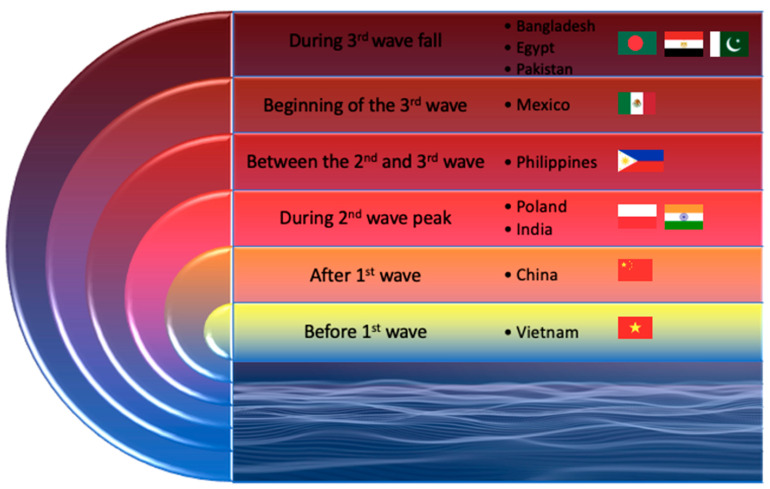
Pandemic point in each country participating in the study. Study start date for individual countries in chronological order: Poland—12 April 2021, China—16 April 2021, Vietnam—26 April 2021, Bangladesh—28 April 2021, India—9 May 2021, Philippines—20 May 2021, Egypt—30 May 2021, Pakistan—1 June 2021, and Mexico—1 July 2021.

The pandemic situation developed in all countries differently. All epidemic information about the COVID-19 pandemic on the first day of conducting the study is presented in Table 1 (Table 1). The wave stages of the pandemic are presented in Figure 1 (Figure 1).

## 5. Results

### 5.1. Sociodemographic Characteristics

#### 5.1.1. Gender and Age

The whole group consisted of 7255 respondents from 9 countries including Poland (n = 1202), Bangladesh (n = 1586), India (n = 484), Mexico (n = 234), Egypt (n = 566), Philippines (n = 2076), Pakistan (n = 506), Vietnam (n = 98) and China (n = 503). Of which, women accounted for 64.95% (n = 4712), and men for 35.05% (n = 2543). Women consist of a majority of participants in the entire study group as well as in individual countries, except for Bangladesh, where the male and female groups were almost equal. The mean age for the whole group was 21.85 ± 3.66 (Me = 21.00) years, where the oldest students were from Bangladesh (M = 23.18 ± 3.27, Me = 23.00), and the youngest from India (M = 19.50 ± 4.03, Me = 19.00) (Supplementary Tables S1, S3 and S34).

#### 5.1.2. Marital Status

Most respondents described their marital status as “single” (86.18%; n = 6252). The highest “informal relationship” status was reported among Polish students (28.12%; n = 338) and “married” by students from China (17.30%; n = 87) (Supplementary Tables S9 and S151).

#### 5.1.3. Having Children

In the entire study group, 96.28% (n = 6985) of students did not have children. Only 2.43% (n = 176) had one child. This observation concerned all countries, especially Poland, where 98.25% (n = 1181) of the respondents had no children. When it comes to having one child, the highest percentage was recorded among Bangladeshi students, equal to 5.23% (n = 83). Whereas the majority of pregnant women during the study were noticed in Pakistan and constituted 3.36% (n = 17) (Supplementary Tables S10, S26, S42 and S122).

#### 5.1.4. Place of Residence

When considering the place of residence in the entire study group, the majority of students lived in cities with more than 600,000 inhabitants, which amounted to 34.31% (n = 2489). On the other hand, the smallest number of respondents came from cities with fewer than 20,000 residents, which accounted for 8.67% (n = 629). In as many as six of the nine examined countries, students came from cities with more than 600,000 residents, this concerned: Pakistan (100%, n = 506), Vietnam (81.63%, n = 80), Mexico (55.56%, n = 130), China (41.55%, n = 209), India (40.08%, n = 194) and the Philippines (28.47%, n = 591). While from Bangladesh (13.37%, n = 212), Philippines (9.87%, n = 205), India (8.88%, n = 43), Poland (6.74%, n = 81) and Vietnam (1.02%, n = 1) the smallest percentage came from small towns (Supplementary Tables S8, S24, S40, S56, S72, S104, S120, S135 and S150).

#### 5.1.5. Housing Situation

Looking at the housing situation of students participating in the survey, it was shown that in the entire study group, 70.39% (n = 5107) lived with parents, while a negligible percentage of 0.43% (n = 31) lived with their children. The overwhelming majority of students in each country stayed in the family home, especially in India (95.45%, n = 462), the Philippines (86.37%, n = 1793), and Mexico (86.32%, n = 202). Only in China, it turned out that the majority of 79.72% (n = 401) lived with roommates. In each country, fewest students lived with their children or with their children and fiancées/partners. Outside Vietnam, where the lowest percentage constituted students living alone (25.51%, n = 25) (Supplementary Tables S11, S59, S75, S107, S137 and S153).

#### 5.1.6. Working Status

As many as 73.51% (n = 5333) of students from the entire analyzed group did not work during the pandemic. The smallest number, 4.18% (n = 303), despite the difficult situation, ran their own businesses. Not having concerned work students from all countries, except those from Vietnam, who most often chose white-collar work (54.08%, n = 53). There was also a slight percentage of students running their own’s business from all countries, except Pakistan (4.55%, n = 23) and Vietnam (2.04%, n = 2), where the least worked physically, and China with 0.99% (n = 5) working mentally (Supplementary Tables S12, S124, S138 and S154).

#### 5.1.7. Field of Study and Year of Study

The vast majority of respondents were medical students 39.13% (n = 2839), followed by social sciences students 20.44% (n = 1483) and the least were those studying technical sciences 12.31% (n = 893). Considering the division of all student groups into particular fields of study, the highest percentage of medical students contained the Indian sample, 96.28% (n = 466). In the case of social and technical sciences, the group of students from the Philippines was mainly made up of those two fields of study, achieving 31.07% (n = 645) and 21.48% (n = 446), respectively. Additionally, the China sample mostly consists of students from artistic or humanistic studies 86.08% (n = 433) (Supplementary Tables S4, S52, S100 and S146). 

#### 5.1.8. Form of Studying

The entire group of students most often had fully online classes during the semester while participating in the study. This situation concerned 59.28% (n = 4299) of students. On the other hand, the smallest percentage had classes completely through direct contact (7.06, n = 512). Exactly the same pattern applied to the Philippines (88.15% vs. 1.54%), India (75.83% vs. 1.03%), Bangladesh (70.55% vs. 2.90%) and Poland (46.42% vs. 2.50%). Only in China, as many as 63.82% (n = 321) of students most often had classes through direct contact, while in Egypt, 9.36% (n = 53) least often had online classes (Supplementary Tables S6, S22, S38, S54, S86, S102 and S148).

#### 5.1.9. Use of Psychological/Psychiatric Services before the Pandemic

An overwhelming number of students from all countries replied that they had not used psychological/psychiatric services before the pandemic (89.26%, n = 6024). In the entire group, only a small percentage of 2.24% (n = 151) used psychiatric services, while 5.97% (n = 403) used psychological services. The vast majority of students in all countries responded in a similar way, except in Mexico, where the proportion of students not using psychological/psychiatric services was only 59.83% (n = 140). There, 31.20% (n = 73) used psychological services (Supplementary Tables S13 and S77). 

#### 5.1.10. Use of Psychological/Psychiatric Services during the Pandemic

Regarding the use of psychological/psychiatric services during the pandemic again, the highest percentage of the entire group, equal to 84.13% (n = 6103), answered that they generally did not use such services. Out of the rest, 6.45% (n = 468) declared to start using the services of a psychologist/psychiatrist because of feeling worse. The fewest respondents declared that they attend visits less often because of the improvement in their well-being (1.08%, n = 78) out of all students (15.87%, n = 1151) that used the help of a psychiatrist or psychologist. Exactly the same observation applies to students from China (96.62% vs. 0.40%), Vietnam (94.90% vs. 1.02%), the Philippines (78.28% vs. 1.40%), Poland (77.37% vs. 0.83%) and Mexico (55.98% vs. 2.14%). Wherein 16.24% (n = 38) of Mexican students admitted that they had to start using psychological or psychiatric services because they felt worse (Supplementary Tables S14, S30, S78, S110, S140 and S156). 

#### 5.1.11. Supplements Intake

In the entire group of respondents, almost half of the students (49.21%, n = 3563) did take supplements during the pandemic. Out of those who took supplements, 25.26% (n = 1829) continued taking the supplements they had taken before the pandemic, 16.85% (n = 1220) started taking them during the pandemic, and only a minor percentage of 7.10% (n = 514) continued supplementation as before and additionally started taking new ones. This was the case in all countries except China, where 0.60% (n = 3) decided to start taking supplements due to the pandemic. Students from the vast majority of countries most often did not take supplements, except for those from Pakistan, Poland, and the Philippines, where they most often continued taking the same supplements as before the outbreak of the pandemic (63.64%, n = 322; 44.19%, n = 529; 32.45%, n = 672, respectively) (Supplementary Tables S15, S31, S111, S126 and S157). 

### 5.2. Attitudes toward Vaccinations

#### 5.2.1. Vaccination against Influenza

In the case of influenza vaccination, as many as 64.48% (n = 4674) of respondents in the entire group were unvaccinated compared to 35.52% (n = 2575) who were vaccinated. Egypt turned out to be the leading country with an exceptionally high percentage of 100% (n = 566) of students not vaccinated against influenza. Only in China (97.21%, n = 488) and Mexico (78.21%, n = 183), the majority constituted vaccinated students (Supplementary Tables S16, S80, S96 and S158). 

#### 5.2.2. Vaccinations against COVID-19

Of all respondents, 22.11% (n = 1604) were vaccinated against COVID-19, as opposed to a great number of students claiming to be unvaccinated, 77.89% (n = 5652). The Pakistani group had the highest rate of vaccinated students at 67.39% (n = 341), and the almost unvaccinated group was that of China with a result of 97.41% (n = 488) (Supplementary Tables S2, S115 and S145) (Table 2).

Poland was the only country where it was mandatory only for medical students to get COVID-19 vaccination, while in Pakistan and Vietnam, it was obligatory for all the students. In no other countries included in this study was a need to get COVID-19 vaccination enabling the students to choose whether to get the vaccine or not. Amongst the medical students who lived in countries where the COVID-19 vaccination was mandatory to attend classes, only 35% wanted to be vaccinated, while 65% did not present such willingness. Since medical students from Poland, Pakistan and Vietnam were obliged to vaccinate against COVID-19, an analysis was made of the reasons given for reluctance to be vaccinated between them and medical students from other countries. The only significant difference in the frequency of reported reasons was that the reluctance to vaccinate was argued less frequently due to the subjective feeling of not belonging to the risk group (7.64 vs. 17.68%, *p* < 0.00001).

### 5.3. Differences in the Examined Variables between the Vaccinated and Unvaccinated Participants

#### 5.3.1. Gender

There was no statistically significant difference between the genders of participants that were vaccinated and unvaccinated in the whole group (*p* = 0.959) and most countries. Only in Pakistan (*p* = 0.033) and China (*p* = 0.001), an important difference could be spotted. In both, female students were more frequently unvaccinated. In Pakistan, 72.48% of males were vaccinated compared to 63.54% of females (*p* = 0.033). As for Chinese students, 7.29% and 1.47% of male and female students underwent vaccination, respectively (*p* = 0.001) (Supplementary Tables S2, S115 and S145). 

#### 5.3.2. Field of Study

In the entire studied group, it was noted that the vaccine coverage rate was related to the field of study (*p* = 0.0001). The students that were the most frequently vaccinated studied medical fields (38.25%), and the least were artistic or humanistic (8.21%). This phenomenon was not found in any other country where the statistical difference was obtained. In Poland, the Philippines, and Pakistan, the highest percentage of vaccinated students was found in the medical field (68.99%, 31.81%, 73.73%, respectively), however the smallest in the science field (1.89%, 5.64%, 42.67%, respectively). Bangladesh students that were the most vaccinated represented social sciences (16.52%), and the least were in technical science (7.83%). Due to significant disproportions in the number of students of particular faculties in China, it was not decided to interpret the obtained results. In India (*p* = 0.222), Mexico (*p* = 0.389), Egypt (*p* = 0.174), and Vietnam (*p* = 0.383) were no significant differences observed taking into account the fields of study (Supplementary Tables S5, S21, S37, S53, S69, S85, S101, S117, S132 and S147). 

#### 5.3.3. Mode of Conducting Classes

The number of vaccinated students differed depending on the way of conducting the classes in the entire group (*p* = 0.00001). The students who were most likely to vaccinate were found to study 30–50% of classes online (39.30%), and the least was found to study completely online (15.14%). In Poland, students with the highest percentage of online classes had the lowest rate of vaccination (8.60%) (*p* = 0.0001). Respectively with the increase in the classes with direct contact, this number increased and reached 83.33% of vaccination in the group with completely direct classes. Chinese students with no online classes showed the smallest percentage of vaccinated students (1.25%) and the highest in students with 30–50% of classes online (10.00%) (*p* = 0.01). In Mexico, due to the small number of students representing some of the classes, the interpretation of the data was not made. Participants from Bangladesh (*p* = 0.267), India (*p* = 0.07), Egypt (*p* = 0.659), the Philippines (*p* = 0.722), Pakistan (*p* = 0.175), and Vietnam (*p* = 0.211) showed no statistically significant differences in vaccine coverage rate depending on the mode of conducting classes (Supplementary Tables S7, S23, S39, S55, S71, S87, S103, S119, S134 and S149).

#### 5.3.4. Willingness to Vaccinate against COVID-19

The majority of the respondents (n = 5025; 69.2%) presented a willingness to be vaccinated while 30.8% of them (n = 2231) did not report such willingness (*p* = 0.00001). The percentage of vaccinated students was statistically different depending on the willingness to get vaccinated (*p* = 0.0001). 98.74% of all participants that did not want to be vaccinated also were unvaccinated. As for students that intended to be vaccinated, 68.64% were not vaccinated. In most countries, all students that did not want to be vaccinated were also unvaccinated (100%), except in Bangladesh (97.94%) and the Philippines (99.48%). The country with the highest number of unvaccinated students that wanted to be vaccinated was Vietnam (85.92%), while the smallest was Pakistan (16.22%). Only Chinese students did not show any statistical difference in intended and being vaccinated (*p* = 0.413) (Supplementary Tables S17, S49, S113, S128, S143 and S159). 

### 5.4. Factors Related to the Willingness to Vaccinate against COVID-19 among Students

#### 5.4.1. Gender

The gender of respondents was found to be associated with the decision to vaccinate against COVID-19 in the case of students from Bangladesh (*p* = 0.00001), Egypt (*p* = 0.00004), and India (*p* = 0.028). The percentage of men who undertake such decisions constituted 50.37%, 70.64%, and 72.68%, respectively. Comparing to the attitude of women in corresponding countries was equal to 39.26%, 53.47%, and 63.10% (Supplementary Tables S34, S50 and S82).

#### 5.4.2. Marital Status

Marital status was significantly related to the willingness or reluctance to COVID-19 vaccination both for the whole group (*p* = 0.0000) as for Pakistani (*p* = 0.00001), Bengali (*p* = 0.007), and Egyptian (*p* = 0.0103) students. 

Alike in the whole study group, Bangladeshi students, who described themselves as in an informal relationship, would prefer to be vaccinated the most (subsequently 73.49% and 52.03%). When considering Pakistani students, such a group represented singles (83.67%), contrariwise to Egyptian students, where married youths indicated utmost readiness to vaccinate (84.62%). Unlike respondents from Bangladesh, married people were the least willing to get vaccinated (32.68%). 

Both in the whole group and among Pakistani students, divorced people wanted to vaccinate the least willingly (correspondingly 42.86% and 14.29%) (Supplementary Tables S9, S41, S89 and S121). 

#### 5.4.3. Having Children

Having children was the variable significantly related to the willingness to vaccinate among students from Pakistan (*p* = 0.00001) and Poland (*p* = 0.009).

Significant differences related to the desire to be vaccinated depending on having children or being pregnant were observed among Pakistani students, amid 94.66% were childless (of which 82.05% wanted to be vaccinated versus 17.95% who did not want) compared to students who had children or were pregnant—5.34% (51.87% vs. 48.13%). The highest percentage of students refusing to be vaccinated depicted the group of pregnant women (44.18% vs. 58.82%) and parents of one child (25% vs. 75%).

Inversely, among Polish students, the most willing to be vaccinated were those who had one child (92.86%) and the least those who did not have children (74.94%). Additionally, 100% of pregnant women did not want to be vaccinated (Supplementary Tables S26 and S122).

#### 5.4.4. Place of Residence

In the case of the entire group (*p* = 0.0000), as well as students of Poland (*p* = 0.0000), Mexico (*p* = 0.0041), Bangladesh (*p* = 0.0142) and India (*p* = 0.0494), a significant relationship was noticed with the place of residence and decision to vaccinate against COVID-19.

In the entire group, students from cities with between 100,000 and 600,000 inhabitants expressed the greatest willingness to vaccinate (76.87%), while those from 20,000 and 100,000 (36.94%) were the lowest. This observation was also visible in the case of students from Bangladesh (correspondingly 49.57% and 38.48%). The same conclusion applied to Polish students, where the highest percentage of respondents wanting to be vaccinated was among those living in cities between 100,000 and 600,000 (84.35%), but the least willing to get vaccinated were those from the countryside (40.82%). Likewise, in Mexico, the highest willingness was found in students living in cities between 20,000 and 100,000 residents (96.43%) versus the villages (62.50%).

In India, the greatest readiness for vaccination was expressed by students from the largest cities with more than 600,000 inhabitants (74.23%), while the lowest by those from small towns with less than 20,000 (44.19%) (Supplementary Tables S8, S40, S56 and S72). 

#### 5.4.5. Housing Situation

There was no relationship between the housing situation and the decision to vaccinate for the entire group, but among Pakistani (*p* = 0.00002), Polish (*p* = 0.00003), and Mexican (*p* = 0.0017) students.

Since Polish students who lived with roommates (83.38%) mostly showed a willingness to vaccination, those who lived with parents (67.95%) were at least determined. Considering Mexican students skeptical about vaccinations were those who lived with a partner or fiancé (42.86%), unlike in Pakistan, where 90.48% of students in the same situation showed readiness to vaccinate. Yet, the least motivated Pakistani students lived with roommates (78.05%) (Supplementary Tables S27, S75 and S123). 

#### 5.4.6. Working Status

Having mental or physical work or running one’s own business showed a relationship with the desire to vaccinate against COVID-19, both among students of the entire study group (*p* = 0.00000) and those from Bangladesh (*p* = 0.00029), Mexico (*p* = 0.0039), Poland (*p* = 0.0195) and Egypt (*p* = 0.055).

In the whole group of respondents, those who worked mentally were most willing to undergo vaccination (71.86%) than those who worked physically (61.35%). The observation refers especially to Polish students; the uttermost percentage of who were willing to be vaccinated was among those working mentally (72.25%) compared to those who worked physically (65.87%). The same consideration applies to Egyptian students (66.67% versus 42.50%).

In Bangladesh, the largest percentage of students who wanted to be vaccinated ran their own businesses (49.54%), and the least worked physically (33.21%). The reverse occurred in the group of Mexican respondents, where 100% of physically working were willing to be vaccinated, contrasted with 92.86% of mentally working students (Supplementary Tables S12, S28, S44, S76 and S92). 

#### 5.4.7. Field of Study

The results of the conducted analysis showed that the field of study determined the willingness or opposition to vaccinating in the entire group of respondents (*p* = 0.0000), as well as in the Polish (*p* = 0.0000), Bengali (*p* = 0.0000), Filipino (*p* = 0.0000) and Pakistani (*p* = 0.0005) group. As in the case of Polish students, the result is not surprising because vaccination was compulsory for medical students, in reference to students from Pakistan, in which all students were obliged to vaccinate—this result is worth emphasizing. 

Alike the entire group, Polish and Bengali medical students most wanted to be vaccinated (73.16%, 85.91%, and 54.17%, respectively). However, in the Philippines, medical students were the least likely to get the vaccine (73.49%). In the case of Polish and Bengali students, those who studied artistic or humanistic studies were the least willing (consecutively 52.99% and 34.03%). Otherwise, artistic, and humanistic Pakistani students showed a supreme willingness to get the vaccine (93.33%), and those studying sciences the least (64.00%), which was also the case with the respondents from all the countries participating in the study (60.63%) (Supplementary Tables S4, S20, S36, S100 and S116). 

#### 5.4.8. Mode of Conducting Classes

The form of studying showed a significant relationship with the willingness to vaccinate for the whole group (*p* = 0.00005), as well as for Poland (*p* = 0.0000), Pakistan (*p* = 0.00004), the Philippines (*p* = 0.0009) and Bangladesh (*p* = 0.008). In the entire group of respondents, students who had more than 50% of online classes (75.58%) wanted to vaccinate the most than those who had up to 30% of online classes (64.88%).

As in the group as a whole, in Bangladesh, the Philippines, and Pakistan, the highest percentage of students who wanted to get vaccinated had more than 50% of online classes (54.62%, 95.00%, 93.84% as follows). On the contrary, in Poland, such a group was made up of students who had up to 30% of online classes (88.57%) compared to those who had classes entirely online (64.70%). In Bangladesh, the smallest percentage of students who wanted to vaccinate had classes completely through direct contact (30.43%), while in the Philippines, those who had classes completely online (82.08%) (Supplementary Tables S6, S22, S38, S102 and S118). 

#### 5.4.9. Use of Psychological/Psychiatric Services before the Pandemic

The use of pre-pandemic psychologist/psychiatrist services showed a significant association with the willingness to vaccinate the entire group (*p* = 0.0168) and Filipino students (*p* = 0.0003).

Interestingly, in the case of the entire group of respondents, students who used both psychological and psychiatric services before the outbreak of the pandemic were most likely to vaccinate themselves (77.78%) in comparison with students who did not use such support at all (31.51%). The situation was quite different among Filipino students—the greatest readiness to vaccinate was reported by students who did not use the services of a psychologist/psychiatrist before the outbreak of the pandemic (84.50%), while the least willing to vaccinate were students who used both the services before the pandemic (30.36%) (Supplementary Tables S13 and S109). 

#### 5.4.10. Use of Psychological/Psychiatric Services during the Pandemic

The use of psychological/psychiatric services during a pandemic was associated with an inclination toward vaccination both for the whole group (*p* = 0.0000) and for students from Pakistan (*p* = 0.0003) and the Philippines (*p* = 0.0198). Considering the group as a whole, the most likely to vaccinate were students who had difficulties contacting a psychologist or a psychiatrist due to a pandemic situation (84.68%), while those who assessed the improvement in well-being despite the pandemic were the least likely to vaccinate (37.18%).

In the Philippines, students who had difficulty contacting a psychologist or psychiatrist (85.71%) also showed the greatest willingness to vaccinate than students who maintained that they continued their visits to a psychologist or psychiatrist with the same frequency as in pre-pandemic times (32.26%). In Pakistan, the largest group that did not want to be vaccinated were those who generally did not use psychological/psychiatric services (22.15%) (Supplementary Tables S14, S110 and S125). 

#### 5.4.11. Supplements Intake

Attitude toward supplements during the pandemic was significantly related to the willingness to vaccinate, both in the whole group (*p* = 0.0000) and in the Philippines (*p* = 0.0000), Bangladesh (*p* = 0.0001), Pakistan (*p* = 0.0043), Mexico (*p* = 0.0303) and Egypt (*p* = 0.0407).

In the overall group of respondents, students who continued taking the supplements they had already taken before the pandemic was most likely to be vaccinated (74.47%) compared to students who did not take any supplements (66.33%). This observation is especially noticeable for Pakistani students (80.43% and 75.68%, respectively). Moreover, in Mexico, 95.35% of students who continued taking pre-pandemic supplements were most eager to get vaccinated.

**Figure 2:**
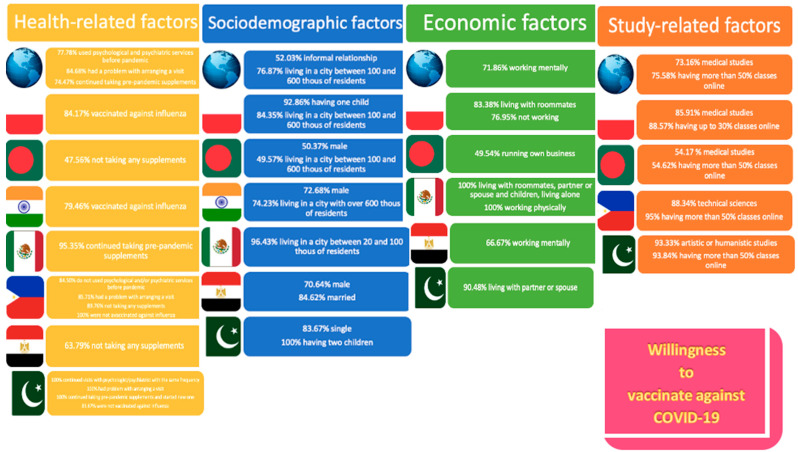
Percentage of students who expressed willingness to vaccinate against COVID-19 concerning only statistically significant results.

The highest percentage of Bangladeshi, Egyptian and Mexican students who refused to vaccinate replied that they began taking dietary supplements during the pandemic (subsequently 68.98%, 57.14%, 23.53%). In both Egypt (63.79%) and the Philippines (89.76%), the most eager students were those who did not take any supplements at all (Supplementary Tables S15, S47, S79, S95, S111 and S126) (Figure 2).

#### 5.4.12. Vaccination against Influenza

Being vaccinated against influenza did not show a significant relationship with the willingness to vaccinate against COVID-19 for the whole group, but it did show a relationship for students from Pakistan (*p* = 0.0000), India (*p* = 0.0000), Philippines (*p* = 0.0000), Mexico (*p* = 0.00001), Bangladesh (*p* = 0.00001) and Poland (*p* = 0.0002).

In the case of students from Poland, Bangladesh, India, and Mexico, those who showed the greatest willingness to be vaccinated against COVID-19 were also vaccinated against influenza with 84.17%, 55.34%, 79.46%, and 97.27%, respectively. The opposite was observed for students from the Philippines and Pakistan; 100% of Filipino students expressing their willingness to vaccinate against COVID-19 were not vaccinated against the flu. In Pakistan, such a group constituted 85.67% of the respondents (Supplementary Tables S32, S48, S64, S80, S112 and S127). 

### 5.5. The Most Common Reasons against COVID-19 Vaccination

The reason why the students did not want to be vaccinated was a closed-ended question with limited multiple-choice options. Of all the answers, 23 reasons were listed (Supplementary Table S160). The most common reason why Polish and Pakistani students refused to vaccinate was: “The vaccine was developed too quickly, there is too little/no evidence of efficacy and safety” (14.90%, 18.18%). In Bangladesh and Mexico: “There is no possibility to be vaccinated right now” (29.51%, 33.33%). In India and Vietnam: “I am not at risk” (16.25%, 18.51%). In Egypt: “I have concerns about the contents of the vaccine” (29.59%). In the Philippines: “The diseases I suffer from” (12.96%), and in China: “Other reason” (34.26%).

Additionally, an analysis was carried out on a group of students who, once vaccinated, decided not to vaccinate further. The most common reasons for such a decision for the entire group of students contained the answers: “I am concerned about the serious side effects of the vaccine,” “I am not at risk,” “The vaccine was developed too quickly, there is too little/no evidence of efficacy and safety,” “The diseases I suffer from,” “I have concerns about the contents of the vaccine,” “There are many vaccine companies on the market, and I don’t know which to trust,” and “I prefer other means of protection against infection.”

## 6. Ethical Considerations

All researchers participating in the study received proper ethical approvals specific to one’s country from the relevant ethical committees before started collecting the responses from students via questionnaires. Before every questionnaire, the respondents had to sign a consent claiming whether they agreed to take part in the study. The informed consent included a brief description and aim of the study, information about the confidentiality and anonymity of the results obtained in the study, as well as the annotation that all of the obtained results will be used only for scientific purposes. Data collected in the study remained confidential and anonymous and were only available to the researchers involved in this study.

## 7. Strengths and Limitations of the Study

Major strengths of our study include a representative population of students (n = 7255) as well as the fact that data were collected from nine countries and four continents (Europe, Africa, Asia, and North America). Further, another positive point of our research is that it was conducted at the beginning of the introduction of the vaccines. Even though its availability differed between the investigated countries, it could clearly present the major reasons for the willingness to vaccinate in the investigated population of students. However, despite the relatively large group of respondents that were successfully collected, our work is not without limitations in terms of inequalities in the number of respondents in particular subgroups. In the whole group of students, the majority was represented by females (64.95%, n = 4712) and medical students (35.05%, n = 2839). Those representations varied through the countries, from 59.9% of females questioned in India to 80.91% in China. Medical students represented 96.28% of Indian respondents compared to 0.40% of China respondents. Additionally, the total number of students was not the same in participating countries. Participants from the Philippines were represented by 2076 students, while Vietnamese by 98. Therefore, it is hard to establish the obtained results as generalizable because the group of students involved in this study was unequal. 

The form of the obtained results had some limitations. The study was conducted in the form of an online self-administered survey where the researchers could not assess the reliability of the information provided by the respondents. Moreover, data were collected using snowball sampling. Not all the social groups and students were reached, and it cannot be ruled out that the most affected could be missed. The students who were involved in this study included only those who were interested in this topic and wanted to contribute to this project. Due to the form, only a limited number of factors were included that could have an impact on students’ willingness to get vaccinated, which were considered. It was justified by creating a sufficiently short survey, which allowed for collecting as many responders as possible.

A different period of data collection, considering the different epidemiological situations in the studied countries, was a certain limitation. Another is that the results reflect only a short period of time, without any insight into longitudinal effects on students’ mental health and changes towards vaccination as they were just introduced. Furthermore, all countries gather the responses at the same time while having various restrictions and epidemiological situations. In addition, we analyzed a limited number of factors that influenced the students’ willingness to vaccinate, which was related to the creation of a sufficiently short questionnaire that would allow us to collect the largest possible group of respondents. Additionally, since we did not know the policy and availability of vaccines against the flu in each of the countries, we did not decide to carry out further in-depth analyzes of this relationship.

## 8. Discussion

When considering the attitude of students toward COVID-19 vaccinations and their willingness to vaccinate, one should recognize the transcultural factor, the time in which the study was set, the severity of the pandemic in the context of undertaken restrictions, new diseases, death, and moods in various countries around the world, but also primarily the fact whether the vaccinations were mandatory or not. Our study aimed to present: (1) the level of vaccination rate against COVID-19 among students from Poland, Bangladesh, India, Mexico, Egypt, the Philippines, Pakistan, Vietnam, and China, (2) factors related to the willingness to vaccinate, considering sociodemographic, economic, health, and study-related factors, and (3) motivations/reasons for reluctance to vaccination. While analyzing the data, we found answers in closed-ended questions with limited multiple-choice options that were strongly culturally related but also those that could be described as universal. As the perception of vaccination has changed dynamically over the decades, more or less scientific opinions have varied, shaping people’s views, the opinion of the young generation during a crisis is worth paying attention to [38]. Moreover, as the course of infection fluctuates from asymptomatic to very serious, the doubts and motives for the decision to vaccinate appeared interesting and, therefore possibly very divergent [39]. Even though many studies have already been conducted on this topic, our study highlights slightly different aspects compared to other similar studies. Firstly, to the best of the authors’ knowledge, it is the first study that collected a such representative number of respondents belonging to the specific group homogenous in terms of age and educational status (in our case, we focused only on the students) involved in the study. The respondents were from nine different countries, while most of the studies primarily focus only on the respondents from one’s country without focusing on the socio-cultural differences and without any aim to make such comparisons. Further, we investigated not only the factors that facilitated one’s willingness to vaccinate but also those factors that made students more reluctant to vaccinate. The last one seems to be extremely important since it provides crucial knowledge regarding students’ fears and worries about the COVID-19 vaccination, which can further be used to educate the students and fill the gaps in knowledge regarding vaccination in general.

Considering the study that we conducted on students in Poland during the first lockdown when the pandemic broke out, we showed that employment status was associated with a general greater severity of emotional distress, as well as depression, anxiety, and stress in relation to people who were not working then. Interestingly, in the current study, the students who were unemployed showed the greatest willingness to be vaccinated. The same situation applied to students who lived with roommates; again, it was the group that constituted the highest percentage of those willing to be vaccinated. Surprisingly, the results of our present study did not show statistical significance in the use of psychological/psychiatric services before or during the pandemic and the preference to vaccinate, despite observations about the severity of emotional difficulties [40].

Compared with our previous studies, the current results showed a completely different phenomenon than at the beginning of the pandemic- namely, that a higher percentage of students use dietary supplements. However, this did not influence the decision to vaccinate against COVID-19 [41].

It should be considered that such a high percentage of unvaccinated students presented in this study might be a result of no compulsion to be vaccinated and no consequences for being unvaccinated. On the other hand, the highest percentage of the vaccinated students in the studied population (who were Pakistani students) was primarily because of the fact that the COVID-19 vaccine was mandatory. This might indicate that even medical students, who, by their attitude and knowledge, should present a group of those who promote vaccinations in the general population, still might be aware of rapid situations such as a quick development of the COVID-19 vaccine. 

When considering the possibility of returning to the traditional/stationary modes of conducting classes at that time, according to Hossain et al., more than half of Bangladeshi students in the study showed hope that the COVID-19 vaccine would bring them back into the classrooms. In our study, Bangladeshi students who had more than 50% of classes online were the most willing to vaccinate. In addition, as in the case of the entire study group and students from Poland, in Bangladesh, the highest percentage of those willing to be vaccinated among all fields of study were medical students [42]. A possible reason for the lower percentage of medical students vaccinated against COVID-19 from the Philippines at that time was related to the two-month difference in vaccination introduction in the country compared to Poland, which took place on 26 December 2020 [43].

The same conclusion as ours about Bengali students was presented by the study by Bari et al.—the highest proportion of those willing to vaccinate came from urban areas. Specifically, in our study, from cities between 100,000 and 600,000 inhabitants [44].

For Indian students, according to a study conducted between November 2020 and January 2021 by Jain L. et al., non-healthcare sector students expressed a greater willingness to be vaccinated than those associated with the medical sector. Our study did not show a statistical relationship between the willingness of Indian students to get vaccinated and the field of study [45]. 

A few months later, Jain J. et al. presented results pointing to about three-quarters of respondents who believed that the vaccine should be compulsory for healthcare professionals and international travelers. Moreover, the study stated that prior adult vaccination did not affect COVID-19 vaccine hesitancy. In our study, a vast percentage of Indian students who expressed the willingness to vaccinate against COVID-19 were also vaccinated against influenza [46]. 

The vaccination situation in the Philippines seems to be difficult, and one of the possible explanations for that might be the fact that the Philippines still remembers the Dengvaxia controversy, which has greatly damaged public confidence in vaccines. Dengvaxia, which was the first dengue vaccine to be approved after years of research, even if it showed significant efficacy in preventing dengue fever, was associated with an increased risk of hospitalization and severe disease among children between 2 to 5 years old [47,48,49]. The Philippines permanently banned Dengvaxia in 2019. A survey conducted by Social Weather Stations (SWS) between April and May 2021 showed significant reluctance to vaccinate against COVID-19. Only about 32% of the Filipino population declared their willingness to vaccinate, and 35% were undecided. It appears the Philippines is still in the stage of building back trust in vaccines [50]. Notwithstanding the depicted situation of distrust, however, our study found that an overwhelming number of Filipino students (83.29%) expressed a desire to be vaccinated against COVID-19, yet 100% of them were not vaccinated against the flu. What should be added is that as of February 2022, 89.34% of higher education institutions’ teaching and non-teacher personnel and 69.23% of tertiary students have been vaccinated against COVID-19 [51].

Other studies also point to the uncertainty of the Filipino population about vaccination and the pandemic itself. Tee et al. proved that 70% of respondents insisted on additional health information about COVID-19. Among others: details on symptoms, advice on prevention and treatment, regular updates for the latest information and for the outbreaks in their local area, advice for people who might need more tailored information, information on the availability and effectiveness of medicine/vaccine for COVID- 19 and updates on the route of transmission of the virus. The study showed that subjects who expected more information about the virus achieved significantly higher anxiety levels than others [52]. 

In a study conducted by El-Monshed et al. during the peak period of Egypt’s COVID-19 pandemic, being married turned out to be an independent factor of greater life satisfaction during a crisis. By contrast, independent predictors of psychological distress were, among others, being female and having a secondary education. We raise this issue because, in our study, being male, married, and working mentally, were significant aspects connected with the desire to vaccinate in the case of Egyptian students. These things considered, working mentally was associated with a willingness to vaccinate against COVID-19 also for the entire group [53]. 

Bukhsh et al. demonstrated the low rate of immunization against influenza in Pakistan by taking the approach of immunizing children as an example. It should be noted that influenza vaccination is not included in the National immunization program of Pakistan. Nevertheless, the presented situation may result from the little knowledge and information on the safety, availability, and significance of the vaccine—as reported by the authors of the study. This is reflected in our results, which show that the overwhelming majority of students who were not vaccinated against influenza, however, expressed a desire to vaccinate against COVID-19 [54].

Furthermore, Shah et al. pointed to significantly higher stress and depression levels in the Pakistani population during the pandemic in an international study, explaining that the local culture relies heavily on family and community involvement. Perhaps hence social isolation and the imposition of various restrictions could have caused emotional difficulties. Interestingly, according to the results of our study, it is the vast majority of people who have children and who live with a partner or spouse expressed their will to vaccinate against COVID-19 [55]. 

Being aware of the complexity of the issues related to making decisions about vaccination against COVID-19 and the fact that the study did not take into account many additional, sometimes difficult to measure, influential variables, e.g., the power of media coverage, education, economic, social, cultural, and religious factors in each country, we limited the generalization of the obtained conclusions in the study.

## 9. Conclusions

The web-based cross-sectional study conducted in nine countries including four continents showed 69.2% of students (n = 5025) presenting willingness to vaccinate against COVID-19, while 22.11% (n = 1604) were already vaccinated. The conducted research indicates transcultural differences in the context of the will to vaccinate depending on the socio-demographic, economic, health, and study-related factors. Noteworthy are those that remained universal for students of all countries participating in the study. The highest percentage of students wishing to be vaccinated were those studying medicine, having more than 50% online classes, who worked mentally, were in informal relationships, living in large cities, between 100,000 and 600,000 inhabitants, used the services of a psychologist and/or psychiatrist before the pandemic, had difficulty with arranging an appointment during the pandemic, and continued taking the same supplements as before the pandemic. Additionally, there were some universal reasons why students who had been vaccinated with one dose refused to vaccinate further. One of the most frequently stated was: “I am concerned about the serious side effects of the vaccine”. Being vaccinated against influenza did not show a significant relationship with the willingness to vaccinate against COVID-19 for the whole group. Our study indicates that knowledge about vaccines, in general, could be improved since students tend to present attitudes that could indicate that they lack even the basic knowledge about the mechanisms of vaccine working, immunology, or basic aspects of public health and the spread of infectious diseases such as SARS-CoV-2 infection. Further, taking into account the vaccine policy in the investigated countries, it seems beneficial to make the COVID-19 vaccination obligatory, as the highest percentage of vaccinated students was found in those countries where the vaccination was mandatory. Such attitudes could potentially provide a safer environment for work and could minimize the spread of the infection.

## Figures and Tables

**Table 1:** The epidemic situation in all countries participating in the study on the first day of survey distribution.

	Population	Total Cases	Daily New Cases	Active Cases	Total Deaths	Daily New Deaths	New Recoveries
**Poland** 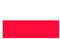	38,265,000	2,586,623	22,966	405,187	54,941	204	32,439
**Bangladesh** 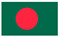	163,187,000	754,614	2955	73,504	11,305	77	N/A
**India** 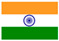	1,339,330,514	17,625,735	319,435	2,879,642	200,751	2845	248,702
**Mexico** 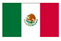	128,600,000	2,519,269	6105	285,692	227,807	61	3149
**Egypt** 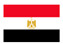	100,000,408	261,666	1007	55,144	15,047	46	N/A
**Philippines** 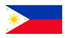	106,651,394	1,165,145	6090	52,000	19,622	116	N/A
**Pakistan** 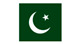	238,181,034	922,824	1771	57,336	20,850	71	3397
**Vietnam** 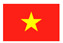	102,789,598	2905	0	361	35	0	N/A
**China** 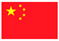	1,411,778,724	90,468	11	299	4636	0	N/A

The information was obtained from the website: https://www.worldometers.info/coronavirus/ (accessed on 10 April 2022) for all countries participating in the study.

**Table 2:** Information on whether vaccines were mandatory, for which students, and the most common vaccine companies used in a particular country at the time of conducting the study.

COVID-19 Vaccine Policy during Conducting the Study			
	Were vaccinations for students compulsory at the time of the study?	Fields of study where it was mandatory to vaccinate	Vaccine company by which students were vaccinated at the time of conducting the study most often
Poland	yes	medical students	Pfizer, Moderna
Bangladesh	no	not applicable	Sinopharm, AstraZeneca, Pfizer, and Moderna were distributed in the different regions.
India	no	not applicable	not applicable
Mexico	no	not applicable	Pfizer for the pediatric population. Pfizer-BioNTech, Cansino, COVAX, AstraZeneca, Sputnik V, Sinovac, Janssen y Moderna The most used one has been AstraZeneca for adults
Egypt	no	not applicable	Sinopharm was the most commonly used vaccine.
Philippines	no	not applicable	Pfizer, moderna, astra zeneca and coronavac in almost equal distribution
Pakistan	yes	medical and all other students whose institutions were open in lockdown	Sinopharm and PakVac
Vietnam	yes	all students	Astra, Pfizer, Moderna
China	no	not applicable	Sinovac

## Data Availability

The data presented in this study are available on request from the corresponding author.

## Supplementary Materials

The following supporting information can be downloaded at: https://www.mdpi.com/article/10.3390/ijerph192114012/s1.

## Abbreviations

COVID-19	Coronavirus Disease 2019
SARS-CoV-2	Severe Acute Respiratory Syndrome Coronavirus-2
