# Supplementary material for: To Vaccinate or Not to Vaccinate—Reasons of Willingness and Reluctance of Students against SARS-CoV-2 Vaccination—An International Experience
Source: Int J Environ Res Public Health. 2022 Oct 27;19(21):14012. doi: 10.3390/ijerph192114012 (PMC9657911; doi:10.3390/ijerph192114012)
Supplement: Supplementary file 1 [file ijerph-19-14012-s001.zip › ijerph-1906903-supplementary.pdf]

Table S1: Willingness to vaccinate in the entire study group during the pandemic.

Gender					
			**Female**	**Male**	**In total**
**Would you like to be vaccinated?**	**Yes**	N	3305	1760	5065
		% of gender	70.14	69.21	
		% of willingness to vaccinate	65.25	34.75	
		% of the group	45.55	24.26	69.81
	**No**	N	1407	783	2190
		% of gender	29.86	30.79	
		% of willingness to vaccinate	64.25	35.75	
		% of the group	19.39	10.79	30.19
	**Total**	N	4712	2543	7255
		% of the entire group	64.95	35.05	100.00
**Chi2 Pearson: 0.6785362, df=1, p=0.41009, N=7255**					

Table S2: Vaccinated students in the entire study group during the pandemic with respect to their gender.

Are you vaccinated against COVID-19?					
			**Yes**	**No**	**In total**
**Gender**	**Female**	N	1041	3672	4713
		% of being vaccinated	64.90	64.97	
		% of gender	22.09	77.91	
	**Male**	N	563	1980	2543
		% of being vaccinated	35.10	35.03	
		% of gender	22.14	77.86	
	**TOTAL**	N	1604	5652	7256
**Chi^2 Pearson: 0.0025, df=1, p=0.959, N=7256**					

Table S3: Age of the students from all of the investigated countries.

	Country	N	Mean	Median	Minimum	Maximum	SD	SE
**Age**	**Entire group**	7274	21.85	21.00	15.00	97.00	3.66	0.04
	**Poland**	1202	22.46	22.00	18.00	51.00	2.67	0.08
	**Banladesh**	1586	23.18	23.00	15.00	55.00	3.27	0.08
	**India**	484	19.50	19.00	17.00	97.00	4.03	0.18
	**Mexico**	234	22.47	21.00	16.00	60.00	6.70	0.44
	**Egypt**	566	20.34	20.00	17.00	26.00	2.10	0.09
	**Philippines**	2076	22.00	21.00	18.00	40.00	4.05	0.09
	**Pakistan**	506	21.12	21.00	16.00	29.00	2.56	0.11
	**Vietnam**	116	21.36	21.00	18.00	30.00	1.84	0.17
	**China**	504	20.11	20.00	15.00	45.00	2.90	0.13

Table S4: Willingness to vaccinate in the entire study group with respect to their field of study during the pandemic.

Field of study								
			**Medical studies**	**Social sciences**	**Technical science**	**Artistic or humanistic studies**	**Sciences**	**In total**
**Would you like to be vaccinated?**	**Yes**	N	2077	1060	642	713	573	5065
		% of field of study	73.16	71.48	71.89	65.11	60.63	
		% of willingness to vaccinate	41.01	20.93	12.68	14.08	11.31	
		% of the group	28.63	14.61	8.85	9.83	7.90	69.81
	**No**	N	762	423	251	382	372	2190
		% of field of study	26.84	28.52	28.11	34.89	39.37	
		% of willingness to vaccinate	34.79	19.32	11.46	17.44	16.99	
		% of the group	10.50	5.83	3.46	5.27	5.13	30.19
	**Total**	N	2839	1483	893	1095	945	7255
		% of the entire group	39.13	20.44	12.31	15.09	13.03	100.00
**Chi^2 Pearson: 68.11336, df=4, p=0.00000, N=7255**								

Table S5: Vaccinated students in the entire study group with respect to their field of study during the pandemic.

Are you vaccinated against COVID-19?					
**Field of study**			**Yes**	**No**	**In total**
	**Medical studies**	N	1086	1753	2839
		% of being vaccinated	67.71	31.02	
		% of field of study	38.25	61.75	
	**Social sciences**	N	239	1244	1483
		% of being vaccinated	14.90	22.01	
		% of field of study	16.12	83.88	
	**Technical science**	N	98	795	893
		% of being vaccinated	6.11	14.07	
		% of field of study	10.97	89.03	
	**Artistic or humanistic studies**	N	90	1006	1096
		% of being vaccinated	5.61	17.80	
		% of field of study	8.21	91.79	
	**Sciences**	N	91	854	945
		% of being vaccinated	5.67	15.11	
		% of field of study	9.63	90.37	
	**TOTAL**		1604	5652	7256
**Chi^2 Pearson: 733.335, df=4, p=0.0000, N=7256**					

Table S6: Willingness to vaccinate in the entire study group with respect to the current types of classes/studying type at university during the pandemic.

How do you currently study at your university (during this semester)?								
			**Completely online**	**More than 50% classes online**	**About 30-50% classes online**	**Up to 30% classes online**	**Completely through direct contact**	**In total**
**Would you like to be vaccinated?**	**Yes**	N	2963	783	579	375	363	5063
		% of studying form	68.92	75.58	70.01	64.88	70.90	
		% of willingness to vaccinate	58.52	15.47	11.44	7.41	7.17	
		% of the group	40.86	10.80	7.98	5.17	5.01	69.82
	**No**	N	1336	253	248	203	149	2189
		% of studying form	31.08	24.42	29.99	35.12	29.10	
		% of willingness to vaccinate	61.03	11.56	11.33	9.27	6.81	
		% of the group	18.42	3.49	3.42	2.80	2.05	30.18
	**Total**	N	4299	1036	827	578	512	7252

		% of the entire group	59.28	14.29	11.40	7.97	7.06	100.00
**Chi² Pearson: 24.94037, df=4, p=0.00005, N=7252**								

Table S7: Vaccinated students in the entire study group with respect to the current types of classes/studying type at university during the pandemic.

**Are you vaccinated against COVID-19?**					
			**Yes**	**No**	**In total**
**Way of conducting classes at the university**	**Completely online**	N	651	3648	4299
		% of being vaccinated	40.61	64.57	
		% of studying form	15.14	84.86	
	**More than 50% classes online**	N	370	667	1037
		% of being vaccinated	23.08	11.81	
		% of studying form	35.68	64.32	
		N	325	502	827

	**About 30-50% classes online**	% of being vaccinated	20.27	8.88	
		% of studying form	39.30	60.70	
	**Up to 30% classes online**	N	168	410	578
		% of being vaccinated	10.48	7.26	
		% of studying form	29.07	70.93	
	**Completely through direct contact**	N	89	423	512
		% of being vaccinated	5.55	7.49	
		% of studying form	17.38	82.62	
	**TOTAL**	N	1603	5650	7253
**Chi² Pearson: 396.921, df= 4, p=0.0000, N=7253**					

Table S8: Willingness to vaccinate in the entire study group with respect to their place of residence during the pandemic.

**Place of residence**							
		**A city with over 600 thous residents**	**A city between 100 and 600 thous residents**	**A city between 20 and 100 thous residents**	**The town below 20 thous residents**	**Village**	**In total**

**Would you like to be vaccinated?**	**Yes**	N	1849	1047	705	418	1046	5065
		% of place of residence	74.29	76.87	63.06	66.45	63.13	
		% of willingness to vaccinate	36.51	20.67	13.92	8.25	20.65	
		% of the the group	25.49	14.43	9.72	5.76	14.42	69.81
	**No**	N	640	315	413	211	611	2190
		% of the place of residence	25.71	23.13	36.94	33.55	36.87	
		% of willingness to vaccinate	29.22	14.38	18.86	9.63	27.90	
		% of the group	8.82	4.34	5.69	2.91	8.42	30.19
	**Total**	N	2489	1362	1118	629	1657	7255
		% of the entire group	34.31	18.77	15.41	8.67	22.84	100.00
**Chi² Pearson: 118.5699, df=4, p=0.0000, N=7255**								

Table S9: Willingness to vaccinate in the entire study group with respect to their marital status during the pandemic.

Marital status							
			**Single**	**Informal relationship**	**Married**	**Divorcee**	**In total**
**Would you like to be vaccinated?**	**Yes**	N	4393	427	239	6	5065
		% of marital status	70.27	73.49	58.58	42.86	
		% of willingness to vaccinate	86.73	8.43	4.72	0.12	
		% of the group	60.55	5.89	3.29	0.08	69.81
	**No**	N	1859	154	169	8	2190
		% of marital status	29.73	26.51	41.42	57.14	
		% of willingness to vaccinate	84.89	7.03	7.72	0.37	
		% of the group	25.62	2.12	2.33	0.11	30.19
	**Total**	N	6252	581	408	14	7255
		% of the entire group	86.18	8.01	5.62	0.19	100.00
**Chi² Pearson: 33.60579, df=3, p=0.00000, N=7255**							

Table S10: Willingness to vaccinate in the entire study group with respect to whether the respondents have children.

			Do you have children?					
			**No**	**I am pregnant or the wife/partner is pregnant**	**One child**	**Two children**	**More than two children**	**In total**
**Would you like to be vaccinated?**	**Yes**	N	4890	18	117	31	8	5065
		% of having children	70.01	50.00	66.48	68.89	72.73	
		% of willingness to vaccinate	96.54	0.36	2.31	0.61	0.16	
		% of the group	67.40	0.25	1.61	0.43	0.11	69.81
	**No**	N	2095	18	59	14	3	2190
		% of having children	29.99	50.00	33.52	31.11	27.27	
		% of willingness to vaccinate	95.66	0.82	2.69	0.64	0.14	
		% of the group	28.88	0.25	0.81	0.19	0.04	30.19
	**Total**	N	6985	36	176	45	11	7255
		% of the entire group	96.28	0.50	2.43	0.62	0.15	100.00
**Chi² Pearson: 10.56777, df=6, p=0.10269, N=7255**								

Table S11: Willingness to vaccinate in the entire study group taking into account with whom the respondents lived during the pandemic.

I live with:									
			Alone	Roommates	Parents	Partner or spouse	Partner or spouse and children	Children	In total
**Would you like to be vaccinated?**	**Yes**	N	316	861	3537	247	82	22	5065
		% of living with	71.82	71.99	69.26	68.99	66.67	70.97	
		% willingness to vaccinate	6.24	17.00	69.83	4.88	1.62	0.43	
		% of the group	4.36	11.87	48.75	3.40	1.13	0.30	69.81
	**No**	N	124	335	1570	111	41	9	2190
		% of living with	28.18	28.01	30.74	31.01	33.33	29.03	
		% of willingness to vaccinate	5.66	15.30	71.69	5.07	1.87	0.41	
		% of the group	1.71	4.62	21.64	1.53	0.57	0.12	30.19
	**Total**	N	440	1196	5107	358	123	31	7255
		% of the entire group	6.06	16.49	70.39	4.93	1.70	0.43	100.00
**Chi² Pearson: 4.987073, df=5, p=0.41746, N=7255**									

Table S12: Willingness to vaccinate in the entire study group with respect to one's work during the pandemic.

Are you currently working?							
			**No, I do not work**	**I work mentally**	**I work physically**	**I run my own business**	**In total**
**Would you like to be vaccinated?**	**Yes**	N	3774	600	481	210	5065
		% of currently working	70.77	71.86	61.35	69.31	
		% of willingness to vaccinate	74.51	11.85	9.50	4.15	
		% of the group	52.02	8.27	6.63	2.89	69.81
	**No**	N	1559	235	303	93	2190
		% of currently working	29.23	28.14	38.65	30.69	
		% of willingness to vaccinate	71.19	10.73	13.84	4.25	
		% of the group	21.49	3.24	4.18	1.28	30.19
	**Total**	N	5333	835	784	303	7255
		% of the entire group	73.51	11.51	10.81	4.18	100.00
**Chi^2 Pearson: 30.62599, df=3, p=0.00000, N=7255**							

Table S13: Willingness to vaccinate in the entire study group with respect to the fact whether one's used psychological/psychiatric services before the pandemic outbreak.

Did you use psychological/psychiatric services before the pandemics broke out?							
			**No**	**Yes, psychological**	**Yes, psychiatric**	**Yes, psychological and psychiatric**	**In total**
**Would you like to be vaccinated?**	**Yes**	N	4126	295	104	133	4658
		% of using services	68.49	73.20	68.87	77.78	
		% of willingness to vaccinate	88.58	6.33	2.23	2.86	
		% of the group	61.13	4.37	1.54	1.97	69.02
	**No**	N	1898	108	47	38	2091
		% of using services	31.51	26.80	31.13	22.22	

		% of willingness to vaccinate	90.77	5.16	2.25	1.82	
		% of the group	28.12	1.60	0.70	0.56	30.98
	**Total**	N	6024	403	151	171	6749
		% of the entire group	89.26	5.97	2.24	2.53	100.00
**Chi² Pearson: 10.21280, df=3, p=0.01684, N=6749**							

Table S14: Willingness to vaccinate in the entire study group with respect to the fact whether one's used psychological/psychiatric services during the pandemic.

**Did you use psychological/psychiatric services during the pandemics?**								
		**I generally do not use the help of a psychologist/psychiatrist**	**I had to start using the services of a psychologist/psychiatrist because I feel worse**	**I continue to use the services with the same**	**I need more frequent visits because**	**I have difficulties with contacting a psychiatrist/psychologist due to the epidemic**	**I go to visits less often due to the improvement**	**In total**

Would you like to be vaccinated?					frequency as before the pandemic	I feel worse		of my well-being	
	Yes	N	4180	358	128	67	282	49	5064
		% of using services	68.49	76.50	72.73	69.79	84.68	62.82	
		% of willingness to vaccinate	82.54	7.07	2.53	1.32	5.57	0.97	
		% of the group	57.62	4.94	1.76	0.92	3.89	0.68	69.81
	No	N	1923	110	48	29	51	29	2190
		% of using services	31.51	23.50	27.27	30.21	15.32	37.18	
		% of willingness to vaccinate	87.81	5.02	2.19	1.32	2.33	1.32	
		% of the group	26.51	1.52	0.66	0.40	0.70	0.40	30.19
	Total	N	6103	468	176	96	333	78	7254
		% of the entire group	84.13	6.45	2.43	1.32	4.59	1.08	100.00
**Chi^2 Pearson: 52.44188, df=5, p=0.00000, N=7254**									

Table S15: Willingness to vaccinate in the entire study group with respect to the fact whether the students were taking any supplements during the pandemic.

Would you like to be vaccinated?			Do you take any supplements during the pandemics?				
			**Yes, I continue to take those which I was taken before the pandemics and those which I've started during the pandemics**	**Yes, I've started to take them during the pandemics**	**Yes, I continue to take those which I was taking before the pandemics**	**No**	**In total**
	**Yes**	N	377	877	1362	2439	5055
		% of taking supplements	73.35	71.89	74.47	66.33	
		% of willingness to vaccinate	7.46	17.35	26.94	48.25	
		% of the group	5.21	12.11	18.81	33.69	69.82
	**No**	N	137	343	467	1238	2185
		% of taking supplements	26.65	28.11	25.53	33.67	
		% of willingness to vaccinate	6.27	15.70	21.37	56.66	
		% of the group	1.89	4.74	6.45	17.10	30.18
	**Total**	N	514	1220	1829	3677	7240
		% of the entire group	7.10	16.85	25.26	50.79	100.00
**Chi² Pearson: 45.48537, df=3, p=0.00000, N=7240**							

Table S16: Willingness to vaccinate in the entire study group with respect to the fact whether the students are being vaccinated against influenza.

**Are you being vaccinated against influenza?**					
**Would you like to be vaccinated?**			**No**	**Yes**	**In total**
	**Yes**	N	3263	1799	5062
		% of being vaccinated	69.81	69.86	
		% of willingness to vaccinate	64.46	35.54	
		% of the group	45.01	24.82	69.83
	**No**	N	1411	776	2187
		% of being vaccinated	30.19	30.14	
		% of willingness to vaccinate	64.52	35.48	
		% of the group	19.46	10.70	30.17
	**Total**	N	4674	2575	7249
		% of the entire group	64.48	35.52	100.00
**Chi^2 Pearson: 0.0021600, df=1, p=0.96293, N=7249**					

Table S17: Willingness to vaccinate in the entire study group with respect to being already vaccinated during the pandemic.

Are you vaccinated against COVID-19?					
**Would you like to be vaccinated?**			**Yes**	**No**	**In total**
	**Yes**	N	1576	3449	5025
		% of being vaccinated	98.25	61.02	
		% of willingness to vaccinate	31.36	68.64	
	**No**	N	28	2203	2231
		% of being vaccinated	1.75	38.98	
		% of willingness to vaccinate	1.26	98.74	
	**TOTAL**	N	1604	5652	7256
**Chi² Pearson: 813.3815, df=1, p=0.0000, N=7256**					

Table S18: Willingness to vaccinate amongst Polish students with respect to their gender.

Gender					
			Female	Male	In total
Would you like to be vaccinated?	Yes	N	695	205	900
		% of gender	73.86	78.54	
		% of willingness to vaccinate	77.22	22.78	
		% of the group	57.82	17.05	74.88
	No	N	246	56	302
		% of gender	26.14	21.46	
		% of willingness to vaccinate	81.46	18.54	
		% of the group	20.47	4.66	25.12
	Total	N	941	261	1202
		% of the entire group	78.29	21.71	100.00
**Chi² Pearson: 2.385479, df=1, p=0.12247, N=1202**					

Table S19: Vaccinated Polish students during the pandemic with respect to their gender.

Are you vaccinated against COVID-19?					
Gender			**Yes**	**No**	**In total**
	**Female**	N	397	544	941
		% of being vaccinated	81.02	76.40	
		% of gender	42.19	57.81	
	**Male**	N	93	168	261
		% of being vaccinated	18.98	23.60	
		% of gender	35.63	64.37	
	**TOTAL**	N	490	712	1202

Chi² Pearson: 3.638, df=1, p=0.056, N=1202

Table S20: Willingness to vaccinate amongst Polish students with respect to their field of study.

Field of study								
			Medical studies	Social sciences	Technical science	Artistic or humanistic studies	Sciences	In total
	**Yes**	N	579	119	71	62	69	900

**Would you like to be vaccinated?**		% of field of study	85.91	64.67	58.68	52.99	65.09	
		% of willingness to vaccinate	64.33	13.22	7.89	6.89	7.67	
		% of the group	48.17	9.90	5.91	5.16	5.74	74.88
	**No**	N	95	65	50	55	37	302
		% of field of study	14.09	35.33	41.32	47.01	34.91	
		% of willingness to vaccinate	31.46	21.52	16.56	18.21	12.25	
		% of the group	7.90	5.41	4.16	4.58	3.08	25.12
	**Total**	N	674	184	121	117	106	1202
		% of the entire group	56.07	15.31	10.07	9.73	8.82	100.00
**Chi² Pearson: 105.8154, df=4, p=0.0000, N=1202**								

Table S21: Vaccinated Polish students with respect to their field of study during the pandemic.

Are you vaccinated against COVID-19?					
			**Yes**	**No**	**In total**
**Field of study**	**Medical studies**	N	465	209	674
		% of being vaccinated	94.90	29.35	
		% of field of study	68.99	31.01	
	**Social sciences**	N	15	169	184
		% of being vaccinated	3.06	23.74	
		% of field of study	8.15	91.85	
	**Technical science**	N	4	117	121
		% of being vaccinated	0.82	16.43	
		% of field of study	3.31	96.69	
	**Artistic or humanistic studies**	N	4	113	117
		% of being vaccinated	0.82	15.87	
		% of field of study	3.42	96.58	
	**Sciences**	N	2	104	106
		% of being vaccinated	0.41	14.61	
		% of field of study	1.89	98.11	
	**TOTAL**	N	490	712	1202
**Chi^2 Pearson: 507.668, df=4, p=0.0000 N=1202**					

Table S22: Willingness to vaccinate amongst Polish students with respect to the current types of classes/studying type at university during the pandemic.

How do you currently study at your university (during this semester)?								
			**Completely online**	**More than 50% classes online**	**About 30-50% classes online**	**Up to 30% classes online**	**Completely through direct contact**	**In total**
**Would you like to be vaccinated?**	**Yes**	N	361	233	187	93	26	900
		% of studying form	64.70	78.98	87.38	88.57	86.67	
		% of willingness to vaccinate	40.11	25.89	20.78	10.33	2.89	
		% of the group	30.03	19.38	15.56	7.74	2.16	74.88
	**No**	N	197	62	27	12	4	302
		% of studying form	35.30	21.02	12.62	11.43	13.33	
		% of willingness to vaccinate	65.23	20.53	8.94	3.97	1.32	
		% of the group	16.39	5.16	2.25	1.00	0.33	25.12
	**Total**	N	558	295	214	105	30	1202
		% of the entire group	46.42	24.54	17.80	8.74	2.50	100.00
**Chi^2 Pearson: 63.86863, df=4, p=0.00000, N=1202**								

Table S23: Vaccinated Polish students with respect to the current types of classes/studying type at university during the pandemic.

Are you vaccinated against COVID-19?					
			**Yes**	**No**	**In total**
**Way of conducting classes at the university**	**Completely online**	N	48	510	558
		% of being vaccinated	9.80	71.63	
		% of studying form	8.60	91.40	
	**More than 50% classes online**	N	170	125	295
		% of being vaccinated	34.69	17.56	
		% of studying form	57.63	42.37	
	**About 30-50% classes online**	N	164	50	214
		% of being vaccinated	33.47	7.02	
		% of studying form	76.64	23.36	
	**Up to 30% classes online**	N	83	22	105
		% of being vaccinated	16.94	3.09	
		% of studying form	79.05	20.95	
	**Completely through direct contact**	N	25	5	30
		% of being vaccinated	5.10	0.70	
		% of studying form	83.33	16.67	
	**TOTAL**	N	490	712	1202
**Chi^2 Pearson: 474.049, df= 4, p=0.0000, N=1202**					

Table S24: Willingness to vaccinate amongst Polish students with respect to their place of residence during the pandemic.

Place of residence								
			**A city with over 600 thous residents**	**A city between 100 and 600 thous residents**	**A city between 20 and 100 thous residents**	**The town below 20 thous residents**	**Village**	**In total**
**Would you like to be vaccinated?**	**Yes**	N	247	345	103	60	145	900
		% of place of residence	80.98	84.35	63.58	74.07	59.18	
		% of willingness to vaccinate	27.44	38.33	11.44	6.67	16.11	
		% of the group	20.55	28.70	8.57	4.99	12.06	74.88
	**No**	N	58	64	59	21	100	302
		% of place of residence	19.02	15.65	36.42	25.93	40.82	
		% of willingness to vaccinate	19.21	21.19	19.54	6.95	33.11	
		% of the group	4.83	5.32	4.91	1.75	8.32	25.12
	**Total**	N	305	409	162	81	245	1202
		% of the entire group	25.37	34.03	13.48	6.74	20.38	100.00
**Chi² Pearson: 68.65600, df=4, p=0.00000, N=1202**								

Table S25: Willingness to vaccinate amongst Polish students with respect to their marital status during the pandemic.

			Marital status				
			**Single**	**Informal relationship**	**Married**	**Divorcee**	**In total**
**Would you like to be vaccinated?**	**Yes**	N	613	268	18	1	900
		% of marital status	73.24	79.29	69.23	100.00	
		% of willingness to vaccinate	68.11	29.78	2.00	0.11	
		% of the group	51.00	22.30	1.50	0.08	74.88
	**No**	N	224	70	8	0	302
		% of marital status	26.76	20.71	30.77	0.00	
		% of willingness to vaccinate	74.17	23.18	2.65	0.00	
		% of the group	18.64	5.82	0.67	0.00	25.12
	**Total**	N	837	338	26	1	1202
		% of the entire group	69.63	28.12	2.16	0.08	100.00
**Chi² Pearson: 5.470583, df=3, p=0.14041, N=1202**							

Table S26: Willingness to vaccinate amongst Polish students with respect to whether the respondents have children.

Do you have children?							
**Would you like to be vaccinated?**			**No**	**I am pregnant or the wife/partner is pregnant**	**One child**	**Two children**	**In total**
	**Yes**	N	885	0	13	1	900
		% of having children	74.94	0.00	92.86	50.00	
		% of willingness to vaccinate	98.33	0.00	1.44	0.11	
		% of the group	73.63	0.00	1.08	0.08	74.88
	**No**	N	296	3	1	1	302

		% of having children	25.06	100.00	7.14	50.00	
		% of willingness to vaccinate	98.01	0.99	0.33	0.33	
		% of the group	24.63	0.25	0.08	0.08	25.12
	**Total**	N	1181	3	14	2	1202
		% of the entire group	98.25	0.25	1.16	0.17	100.00
**Chi² Pearson: 15.32265, df=5, p=0.00907, N=1202**							

Table S27: Willingness to vaccinate amongst Polish taking into account with whom the respondents lived during the pandemic.

**I live with:**									
			**Alone**	**Roommates**	**Parents**	**Partner or spouse**	**Partner or spouse and children**	**Children**	**In total**
	**Yes**	N	111	286	354	139	9	1	900

**Would you like to be vaccinated?**		% of living with	78.17	83.38	67.95	76.80	69.23	50.00	
		% of willingness to vaccinate	12.33	31.78	39.33	15.44	1.00	0.11	
		% of the group	9.23	23.79	29.45	11.56	0.75	0.08	74.88
	**No**	N	31	57	167	42	4	1	302
		% of living with	21.83	16.62	32.05	23.20	30.77	50.00	
		% of willingness to vaccinate	10.26	18.87	55.30	13.91	1.32	0.33	
		% of the group	2.58	4.74	13.89	3.49	0.33	0.08	25.12
	**Total**	N	142	343	521	181	13	2	1202
		% of the entire group	11.81	28.54	43.34	15.06	1.08	0.17	100.00
**Chi^2 Pearson: 28.54212, df=5, p=0.00003, N=1202**									

Table S28: Willingness to vaccinate amongst Polish students with respect to one's work during the pandemic.

			**Are you currently working?**				
**Would you like to be vaccinated?**			**No, I do not work**	**I work mentally**	**I work physically**	**I run my own business**	**In total**
	**Yes**	N	681	125	83	11	900
		% of currently working	76.95	72.25	65.87	61.11	
		% of willingness to vaccinate	75.67	13.89	9.22	1.22	
		% of the group	56.66	10.40	6.91	0.92	74.88
	**No**	N	204	48	43	7	302
		% of currently working	23.05	27.75	34.13	38.89	
		% of willingness to vaccinate	67.55	15.89	14.24	2.32	
		% of the group	16.97	3.99	3.58	0.58	25.12
	**Total**	N	885	173	126	18	1202
		% of the entire group	73.63	14.39	10.48	1.50	100.00
			**Chi^2 Pearson: 9.895692, df=3, p=0.01947, N=1202**				

Table S29: Willingness to vaccinate amongst Polish students with respect to the fact whether one's used psychological/psychiatric services before the pandemic outbreak.

Did you use psychological/psychiatric services before the pandemics broke out?							
			**No**	**Yes, psychological**	**Yes, psychiatric**	**Yes, psychological and psychiatric**	**In total**
**Would you like to be vaccinated?**	**Yes**	N	713	83	35	69	900
		% of using services	73.58	77.57	81.40	83.13	
		% of willingness to vaccinate	79.22	9.22	3.89	7.67	
		% of the group	59.32	6.91	2.91	5.74	74.88
	**No**	N	256	24	8	14	302
		% of using services	26.42	22.43	18.60	16.87	
		% of willingness to vaccinate	84.77	7.95	2.65	4.64	
			21.30	2.00	0.67	1.16	25.12
	**Total**	N	969	107	43	83	1202
		% of the entire group	80.62	8.90	3.58	6.91	100.00
**Chi² Pearson: 5.255804, df=3, p=0.15400, N=1202**							

Table S30: Willingness to vaccinate amongst Polish students with respect to the fact whether one's used psychological/psychiatric services during the pandemic.

Would you like to be vaccinated?			I generally do not use the help of a psychologist/psychiatrist	I had to start using the services of a psychologist/psychiatrist because I feel worse	I continue to use the services with the same frequency as before the pandemic	I need more frequent visits because I feel worse	I have difficulties with contacting a psychiatrist/psychologist due to the epidemic	I go to visits less often due to the improvement of my well-being	In total
			**Did you use psychological/psychiatric services during the pandemics?**						
	**Yes**	N	681	121	43	20	25	10	900
		% of using services	73.23	83.45	75.44	76.92	73.53	100.00	
		% of willingness to vaccinate	75.67	13.44	4.78	2.22	2.78	1.11	
		% of the group	56.66	10.07	3.58	1.66	2.08	0.83	74.88
	**No**	N	249	24	14	6	9	0	302
		% of using services	26.77	16.55	24.56	23.08	26.47	0.00	
		% of willingness to vaccinate	82.45	7.95	4.64	1.99	2.98	0.00	

		% of the group	20.72	2.00	1.16	0.50	0.75	0.00	25.12
	**Total**	N	930	145	57	26	34	10	1202
		% of the entire group	77.37	12.06	4.74	2.16	2.83	0.83	100.00
**Chi² Pearson: 10.46579, df=5, p=0.06306, N=1202**									

Table S31: Willingness to vaccinate amongst Polish students with respect to the fact whether the students were taking any supplements during the pandemic.

**Do you take any supplements during the pandemics?**							
**Would you like to be vaccinated?**			**Yes, I continue to take those which I was taken before the pandemics and those which I've started during the pandemics**	**Yes, I've started to take them during the pandemics**	**Yes, I continue to take those which I was taking before the pandemics**	**No**	**In total**
	**Yes**	N	104	149	401	242	896

		% of taking supplements	72.73	77.20	75.80	72.89	
		% of willingness to vaccinate	11.61	16.63	44.75	27.01	
		% of the group	8.69	12.45	33.50	20.22	74.85
	**No**	N	39	44	128	90	301
		% of taking supplements	27.27	22.80	24.20	27.11	
		% of willingness to vaccinate	12.96	14.62	42.52	29.90	
		% of the group	3.26	3.68	10.69	7.52	25.15
	**Total**	N	143	193	529	332	1197
		% of the entire group	11.95	16.12	44.19	27.74	100.00
**Chi² Pearson: 1.841525, df=3, p=0.60594, N=1197**							

Table S32: Willingness to vaccinate amongst Polish students with respect to the fact whether the students are being vaccinated against influenza.

**Are you being vaccinated against influenza?**					
			**No**	**Yes**	**In total**
	**Yes**	N	698	202	900
		% of being vaccinated	72.56	84.17	

		% of willingness to vaccinate	77.56	22.44	
		% of the group	58.07	16.81	74.88
**Would you like to be vaccinated?**	**No**	N	264	38	302
		% of being vaccinated	27.44	15.83	
		% of willingness to vaccinate	87.42	12.58	
		% of the group	21.96	3.16	25.12
	**Total**	N	962	240	1202
		% of the entire group	80.03	19.97	100.00
**Chi² Pearson: 13.76157, df=1, p=0.00021, N=1202**					

Table S33: Willingness to vaccinate amongst Polish students with respect to being already vaccinated during the pandemic.

Are you vaccinated against COVID-19?					
			**Yes**	**No**	**In total**
**Would you like to be vaccinated?**	**Yes**	N	490	410	900
		% of being vaccinated	100.00	57.58	
		% of willingness to vaccinate	54.44	45.56	
	**No**	N	0	302	302
		% of being vaccinated	0.00	42.42	
		% of willingness to vaccinate	0.00	100.00	
	**TOTAL**	N	490	712	1202
**Chi^2 Pearson: 277.578, df=1, p=0.0000, N=1202**					

Table S34: Willingness to vaccinate amongst Bengali students with respect to their gender.

Gender					
Would you like to be vaccinated?			**Female**	**Male**	**In total**
	**Yes**	N	307	405	712
		% of gender	39.26	50.37	
		% of willingness to vaccinate	43.12	56.88	
		% of the group	19.36	25.54	44.89
	**No**	N	475	399	874
		% of gender	60.74	49.63	
		% of willingness to vaccinate	54.35	45.65	
		% of the group	29.95	25.16	55.11
	**Total**	N	782	804	1586
		% of the entire group	49.31	50.69	100.00
**Chi^2 Pearson: 19.79610, df=1, p=0.00001, N=1586**					

Table S35: Vaccinated Bengali students during the pandemic with respect to their gender.

Are you vaccinated against COVID-19?					
Gender			**Yes**	**No**	**In total**
	**Female**	N	103	679	782
		% of being vaccinated	50.49	49.13	
		% of gender	13.17	86.83	
	**Male**	N	101	703	804
		% of being vaccinated	49.51	50.87	
		% of gender	12.56	87.44	
	**TOTAL**	N	204	1382	1586
**Chi^2 Pearson: 0.131, df=1, p=0.717, N= 1586**					

Table S36: Willingness to vaccinate amongst Bengali students with respect to their field of study.

Field of study								
			**Medical studies**	**Social sciences**	**Technical science**	**Artistic or humanistic studies**	**Sciences**	**In total**
**Would you like to be vaccinated?**	**Yes**	N	286	165	70	65	126	712
		% of field of study	54.17	47.01	42.17	34.03	36.00	
		% of willingness to vaccinate	40.17	23.17	9.83	9.13	17.70	
		% of the group	18.03	10.40	4.41	4.10	7.94	44.89
	**No**	N	242	186	96	126	224	874
		% of field of study	45.83	52.99	57.83	65.97	64.00	
		% of willingness to vaccinate	27.69	21.28	10.98	14.42	25.63	
		% of the group	15.26	11.73	6.05	7.94	14.12	55.11
	**Total**	N	528	351	166	191	350	1586
		% of the entire group	33.29	22.13	10.47	12.04	22.07	100.00
**Chi² Pearson: 39.78484, df=4, p=0.00000, N=1586**								

Table S37: Vaccinated Bengali students with respect to their field of study during the pandemic.

Are you vaccinated against COVID-19?					
			**Yes**	**No**	**In total**
**Field of study**	**Medical studies**	N	73	455	528
		% of being vaccinated	35.78	32.92	
		% of field of study	13.83	86.17	
	**Social sciences**	N	58	293	351
		% of being vaccinated	28.43	21.20	
		% of field of study	16.52	83.48	
	**Technical science**	N	13	153	166
		% of being vaccinated	6.37	11.07	
		% of field of study	7.83	92.17	
	**Artistic or humanistic studies**	N	31	160	191
		% of being vaccinated	15.20	11.58	
		% of field of study	16.23	83.77	
	**Sciences**	N	29	321	350
		% of being vaccinated	14.22	23.23	
		% of field of study	8.29	91.71	
	**TOTAL**	N	204	1382	1586

Chi² Pearson: 16.859, df=4, p=0.002, N=1586

Table S38: Willingness to vaccinate amongst Bengali students with respect to the current types of classes/studying type at university during the pandemic.

How do you currently study at your university (during this semester)?								
			Completely online	More than 50% classes online	About 30-50% classes online	Up to 30% classes online	Completely through direct contact	In total
Would you like to be vaccinated?	Yes	N	513	65	56	64	14	712
		% of studying form	45.84	54.62	44.09	36.57	30.43	
		% of willingness to vaccinate	72.05	9.13	7.87	8.99	1.97	
		% of the group	32.35	4.10	3.53	4.04	0.88	44.89
	No	N	606	54	71	111	32	874
		% of studying form	54.16	45.38	55.91	63.43	69.57	
		% of willingness to vaccinate	69.34	6.18	8.12	12.70	3.66	
		% of the group	38.21	3.40	4.48	7.00	2.02	55.11

	Total	N	1119	119	127	175	46	1586
		% of the entire group	70.55	7.50	8.01	11.03	2.90	100.00
**Chi² Pearson: 13.78051, df=4, p=0.00803, N=1586**								

Table S39: Vaccinated Bengali students with respect to the current types of classes/studying type at university during the pandemic.

**Are you vaccinated against COVID-19?**					
			**Yes**	**No**	**In total**
**Way of conducting classes at the university**	**Completely online**	N	143	976	1119
		% of being vaccinated	70.10	70.62	
		% of studying form	12.78	87.22	
	**More than 50% classes online**	N	21	98	119
		% of being vaccinated	10.29	7.09	
		% of studying form	17.65	82.35	

	**About 30-50% classes online**	N	19	108	127
		% of being vaccinated	9.31	7.81	
		% of studying form	14.96	85.04	
	**Up to 30% classes online**	N	17	158	175
		% of being vaccinated	8.33	11.43	
		% of studying form	9.71	90.29	
	**Completely through direct contact**	N	4	42	46
		% of being vaccinated	1.96	3.04	
		% of studying form	8.70	91.30	
	**TOTAL**	N	204	1382	1586
**Chi^2 Pearson: 5.196, df=4, p=0.267, N= 1586**					

Table S40: Willingness to vaccinate amongst Bengali students with respect to their place of residence.

**Place of residence**							
		**A city with over 600 thous residents**	**A city between 100 and 600 thous residents**	**A city between 20 and 100**	**The town below 20 thous residents**	**Village**	**In total**

					thous residents			
**Would you like to be vaccinated?**	**Yes**	N	117	115	152	92	236	712
		% of place of residence	43.82	49.57	38.48	43.40	49.17	
		% of willingness to vaccinate	16.43	16.15	21.35	12.92	33.15	
		% of the group	7.38	7.25	9.58	5.80	14.88	44.89
	**No**	N	150	117	243	120	244	874
		% of place of residence	56.18	50.43	61.52	56.60	50.83	
		% of willingness to vaccinate	17.16	13.39	27.80	13.73	27.92	
		% of the group	9.46	7.38	15.32	7.57	15.38	55.11
	**Total**	N	267	232	395	212	480	1586
		% of the entire study group	16.83	14.63	24.91	13.37	30.26	100.00
**Chi² Pearson: 12.47476, df=4, p=0.01415, N=1586**								

Table S41: Willingness to vaccinate amongst Bengali students with respect to their marital status during the pandemic.

Marital status							
			**Single**	**Informal relationship**	**Married**	**Divorcee**	**In total**
**Would you like to be vaccinated?**	**Yes**	N	597	64	50	1	712
		% of marital status	45.64	52.03	32.68	50.00	
		% of willingness to vaccinate	83.85	8.99	7.02	0.14	
		% of the group	37.64	4.04	3.15	0.06	44.89
	**No**	N	711	59	103	1	874
		% of marital status	54.36	47.97	67.32	50.00	
		% of willingness to vaccinate	81.35	6.75	11.78	0.11	
		% of the group	44.83	3.72	6.49	0.06	55.11
	**Total**	N	1308	123	153	2	1586
		% of the entire group	82.47	7.76	9.65	0.13	100.00
**Chi² Pearson: 12.07723, df=3, p=0.00712, N=1586**							

Table S42: Willingness to vaccinate amongst Bengali students with respect to whether the respondents have children.

			Do you have children?					
			**No**	**I am pregnant or the wife/partner is pregnant**	**One child**	**Two children**	**More than two children**	**In total**
**Would you like to be vaccinated?**	**Yes**	N	670	0	36	4	2	712
		% of having children	45.03	0.00	43.37	44.44	50.00	
		% of willingness to vaccinate	94.10	0.00	5.06	0.56	0.28	
		% of the group	42.24	0.00	2.27	0.25	0.13	44.89
	**No**	N	818	2	47	5	2	874
		% of having children	54.97	100.00	56.63	55.56	50.00	
		% of willingness to vaccinate	93.59	0.23	5.38	0.57	0.23	
		% of the group	51.58	0.13	2.96	0.32	0.13	55.11
	**Total**	N	1488	2	83	9	4	1586
		% of the entire group	93.82	0.13	5.23	0.57	0.25	100.00
**Chi² Pearson: 1.760451, df=4, p=0.77971, N=1586**								

Table S43: Willingness to vaccinate amongst Bengali students taking into account with whom the respondents lived during the pandemic.

I live with:									
			**Alone**	**Roommates**	**Parents**	**Partner or spouse**	**Partner or spouse and children**	**Children**	**In total**
**Would you like to be vaccinated?**	**Yes**	N	72	98	502	24	11	5	712
		% of living with	51.43	48.51	44.39	32.43	39.29	45.45	
		% of willingness to vaccinate	10.11	13.76	70.51	3.37	1.54	0.70	
		% of the group	4.54	6.18	31.65	1.51	0.69	0.32	44.89
	**No**	N	68	104	629	50	17	6	874
		% of living with	48.57	51.49	55.61	67.57	60.71	54.55	
		% of willingness to vaccinate	7.78	11.90	71.97	5.72	1.95	0.69	
			4.29	6.56	39.66	3.15	1.07	0.38	55.11
	**Total**	N	140	202	1131	74	28	11	1586
		% of the entire group	8.83	12.74	71.31	4.67	1.77	0.69	100.00
**Chi^2 Pearson: 8.607611, df=5, p=0.12578, N=1586**									

Table S44: Willingness to vaccinate amongst Bengali students with respect to one's work during the pandemic.

			No, I do not work	I work mentally	I work physically	I run my own business	In total
			**Are you currently working?**				
**Would you like to be vaccinated?**	**Yes**	N	514	55	89	54	712
		% of currently working	47.50	43.31	33.21	49.54	
		% of willingness to vaccinate	72.19	7.72	12.50	7.58	
		% of the group	32.41	3.47	5.61	3.40	44.89
	**No**	N	568	72	179	55	874
		% of currently working	52.50	56.69	66.79	50.46	
		% of willingness to vaccinate	64.99	8.24	20.48	6.29	
		% of the group	35.81	4.54	11.29	3.47	55.11
	**Total**	N	1082	127	268	109	1586
		% of the entire group	68.22	8.01	16.90	6.87	100.00

Chi² Pearson: 18.85307, df=3, p=0.00029, N=1586

Table S45: Willingness to vaccinate amongst Bengali students with respect to the fact whether one's used psychological/psychiatric services before the pandemic outbreak.

Did you use psychological/psychiatric services before the pandemics broke out?							
			**No**	**Yes, psychological**	**Yes, psychiatric**	**Yes, psychological and psychiatric**	**In total**
**Would you like to be vaccinated?**	**Yes**	N	678	28	2	4	712
		% of using services	45.29	42.42	15.38	40.00	
		% of willingness to vaccinate	95.22	3.93	0.28	0.56	
		% of the group	42.75	1.77	0.13	0.25	44.89

	No	N	819	38	11	6	874
		% of using services	54.71	57.58	84.62	60.00	
		% of willingness to vaccinate	93.71	4.35	1.26	0.69	
		% of the group	51.64	2.40	0.69	0.38	55.11
	Total	N	1497	66	13	10	1586
		% of the entire group	94.39	4.16	0.82	0.63	100.00
**Chi² Pearson: 4.930636, df=3, p=0.17695, N=1586**							

Table S46: Willingness to vaccinate amongst Bengali students with respect to the fact whether one's used psychological/psychiatric services during the pandemic.

**Did you use psychological/psychiatric services during the pandemics?**								
		**I generally do not use the help of a psychologist/psychiatrist**	**I had to start using the services of a**	**I continue to use the services**	**I need more frequent**	**I have difficulties with contacting a**	**I go to visits less often due to the**	**In total**

Would you like to be vaccinated?				psychologist/psychiatrist because I feel worse	with the same frequency as before the pandemic	visits because I feel worse	psychiatrist/psychologist due to the epidemic	improvement of my well-being	
	**Yes**	N	613	62	12	6	10	8	711
		% of using services	43.91	57.94	46.15	35.29	58.82	36.36	
		% of willingness to vaccinate	86.22	8.72	1.69	0.84	1.41	1.13	
		% of the group	38.68	3.91	0.76	0.38	0.63	0.50	44.86
	**No**	N	783	45	14	11	7	14	874
		% of using services	56.09	42.06	53.85	64.71	41.18	63.64	
		% of willingness to vaccinate	89.59	5.15	1.60	1.26	0.80	1.60	
		% of the group	49.40	2.84	0.88	0.69	0.44	0.88	55.14
	**Total**	N	1396	107	26	17	17	22	1585
		% of the entire group	88.08	6.75	1.64	1.07	1.07	1.39	100.00
**Chi² Pearson: 10.54186, df=5, p=0.06126, N=1585**									

Table S47: Willingness to vaccinate amongst Bengali students with respect to the fact whether the students were taking any supplements during the pandemic.

Would you like to be vaccinated?			Do you take any supplements during the pandemics?				
			**Yes, I continue to take those which I was taken before the pandemics and those which I've started during the pandemics**	**Yes, I've started to take them during the pandemics**	**Yes, I continue to take those which I was taking before the pandemics**	**No**	**In total**
	**Yes**	N	13	67	57	575	712
		% of taking suplements	44.83	31.02	43.18	47.56	
		% of willingness to vaccinate	1.83	9.41	8.01	80.76	
		% of the group	0.82	4.22	3.59	36.25	44.89
	**No**	N	16	149	75	634	874
		% of taking supplements	55.17	68.98	56.82	52.44	
		% of willingness to vaccinate	1.83	17.05	8.58	72.54	
		% of the group	1.01	9.39	4.73	39.97	55.11
	**Total**	N	29	216	132	1209	1586
		% of the entire group	1.83	13.62	8.32	76.23	100.00
**Chi² Pearson: 20.43973, df=3, p=0.00014, N=1586**							

Table S48: Willingness to vaccinate amongst Bengali students with respect to the fact whether the students are being vaccinated against influenza.

Are you being vaccinated against influenza?					
			**No**	**Yes**	**In total**
**Would you like to be vaccinated?**	**Yes**	N	513	197	710
		% of being vaccinated	41.88	55.34	
		% of willingness to vaccinate	72.25	27.75	
		% of the group	32.45	12.46	44.91
	**No**	N	712	159	871
		% of being vaccinated	58.12	44.66	
		% of willingness to vaccinate	81.75	18.25	
		% of the group	45.03	10.06	55.09
	**Total**	N	1225	356	1581
		% of the entire group	77.48	22.52	100.00
**Chi² Pearson: 20.19766, df=1, p=0.00001, N=1581**					

Table S49: Willingness to vaccinate amongst Bengali students with respect to being already vaccinated during the pandemic.

Are you vaccinated against COVID-19?					
**Would you like to be vaccinated?**			**Yes**	**No**	**In total**
	**Yes**	N	186	526	712
		% of being vaccinated	91.18	38.06	
		% of willingness to vaccinate	26.12	73.88	
	**No**	N	18	856	874
		% of being vaccinated	8.82	61.94	
		% of willingness to vaccinate	2.06	97.94	
	**TOTAL**	N	204	1382	1586
**Chi² Pearson: 202.7195, df=1, p=0.0000, N=1586**					

Table S50: Willingness to vaccinate amongst Indian students with respect to their gender.

Gender					
Would you like to be vaccinated?			Female	Male	In total
	Yes	N	183	141	324
		% of gender	63.10	72.68	
		% of willingness to vaccinate	56.48	43.52	
		% of the group	37.81	29.13	66.94
	No	N	107	53	160
		% of gender	36.90	27.32	
		% of willingness to vaccinate	66.88	33.13	
		% of the group	22.11	10.95	33.06
	Total	N	290	194	484

		% of the entire group	59.92	40.08	100.00
**Chi² Pearson: 4.817657, df=1, p=0.02817, N=484**					

Table S51: Vaccinated Indian students during the pandemic with respect to their gender.

**Are you vaccinated against COVID-19?**					
**Gender**			**Yes**	**No**	**In total**
	**Female**	N	79	211	290
		% of being vaccinated	53.74	62.61	
		% of gender	27.24	72.76	
	**Male**	N	68	126	194
		% of being vaccinated	46.26	37.39	

		% of gender	35.05	64.95	
	**TOTAL**	N	147	337	484
**Chi[2] Pearson: 3.352, df=1, p=0.067, N=484**					

Table S52: Willingness to vaccinate amongst Indian students with respect to their field of study.

**Field of study**

			Medical studies	Social sciences	Technical science	Artistic or humanistic studies	Sciences	In total
**Would you like to be vaccinated?**	**Yes**	N	314	1	7	2	0	324
		% of field of study	67.38	33.33	77.78	40.00	0.00	
		% of willingness to vaccinate	96.91	0.31	2.16	0.62	0.00	
		% of the group	64.88	0.21	1.45	0.41	0.00	66.94
	**No**	N	152	2	2	3	1	160
		% of field of study	32.62	66.67	22.22	60.00	100.00	
		% of willingness to vaccinate	95.00	1.25	1.25	1.88	0.63	
		% of the group	31.40	0.41	0.41	0.62	0.21	33.06
	**Total**	N	466	3	9	5	1	484
		% of the entire group	96.28	0.62	1.86	1.03	0.21	100.00
**Chi^2 Pearson: 5.714576, df=4, p=0.22150, N=484**								

Table S53: Vaccinated Indian students with respect to their field of study during the pandemic.

Are you vaccinated against COVID-19?					
**Field of study**			**Yes**	**No**	**In total**
	**Medical studies**	N	146	320	466
		% of being vaccinated	99.32	94.96	
		% of field of study	31.33	68.67	
	**Social sciences**	N	0	3	3
		% of being vaccinated	0.00	0.89	
		% of field of study	0.00	100.00	
	**Technical science**	N	1	8	9
		% of being vaccinated	0.68	2.37	
		% of field of study	11.11	88.89	
	**Artistic or humanistic studies**	N	0	5	5
		% of being vaccinated	0.00	1.48	
		% of field of study	0.00	100.00	
	**Sciences**	N	0	1	1
		% of being vaccinated	0.00	0.30	
		% of field of study	0.00	100.00	
	**TOTAL**	N	147	337	484
**Chi² Pearson: 5.707, df=4, p=0.222, N=484**					

Table S54: Willingness to vaccinate amongst Indian students with respect to the current types of classes/studying type at university during the pandemic.

How do you currently study at your university (during this semester)?								
			Completely online	More than 50% classes online	About 30-50% classes online	Up to 30% classes online	Completely through direct contact	In total
**Would you like to be vaccinated?**	**Yes**	N	244	55	14	6	5	324
		% of studying form	66.49	66.27	66.67	75.00	100.00	
		% of willingness to vaccinate	75.31	16.98	4.32	1.85	1.54	
		% of the group	50.41	11.36	2.89	1.24	1.03	66.94
	**No**	N	123	28	7	2	0	160
		% of studying form	33.51	33.73	33.33	25.00	0.00	
		% of willingness to vaccinate	76.88	17.50	4.38	1.25	0.00	
		% of the group	25.41	5.79	1.45	0.41	0.00	33.06
	**Total**	N	367	83	21	8	5	484
		% of the entire group	75.83	17.15	4.34	1.65	1.03	100.00
**Chi^2 Pearson: 2.756429, df=4, p=0.59938, N=484**								

Table S55: Vaccinated Indian students with respect to the current types of classes/studying type at university during the pandemic.

**Are you vaccinated against COVID-19?**					
**Way of conducting classes at the university**			**Yes**	**No**	**In total**
	**Completely online**	N	103	264	367
		% of being vaccinated	70.07	78.34	
		% of studying form	28.07	71.93	
	**More than 50% classes online**	N	27	56	83
		% of being vaccinated	18.37	16.62	
		% of studying form	32.53	67.47	
	**About 30-50% classes online**	N	9	12	21
		% of being vaccinated	6.12	3.56	
		% of studying form	42.86	57.14	
	**Up to 30% classes online**	N	5	3	8
		% of being vaccinated	3.40	0.89	
		% of studying form	62.50	37.50	
	**Completely through direct contact**	N	3	2	5
		% of being vaccinated	2.04	0.59	
		% of studying form	60.00	40.00	
	**TOTAL**	N	147	337	484
**Chi^2 Pearson: 8.634, df=4, p=0.07, N= 484**					

Table S56: Willingness to vaccinate amongst Indian students with respect to their place of residence during the pandemic.

			Place of residence					
			**A city with over 600 thous residents**	**A city between 100 and 600 thous residents**	**A city between 20 and 100 thous residents**	**The town below 20 thous residents**	**Village**	**In total**
**Would you like to be vaccinated?**	**Yes**	N	144	58	50	24	48	324
		% of place of residence	74.23	64.44	65.79	55.81	59.26	
		% of willingness to vaccinate	44.44	17.90	15.43	7.41	14.81	
		% of the group	29.75	11.98	10.33	4.96	9.92	66.94
	**No**	N	50	32	26	19	33	160
		% of place of residence	25.77	35.56	34.21	44.19	40.74	
		% of willingness to vaccinate	31.25	20.00	16.25	11.88	20.63	
		% of the group	10.33	6.61	5.37	3.93	6.82	33.06
	**Total**	N	194	90	76	43	81	484
		% of the entire group	40.08	18.60	15.70	8.88	16.74	100.00
**Chi^2 Pearson: 9.518204, df=4, p=0.0493, N=484**								

Table S57: Willingness to vaccinate amongst Indian students with respect to their marital status during the pandemic.

Marital status						
			**Single**	**Informal relationship**	**Married**	**In total**
**Would you like to be vaccinated?**	**Yes**	N	321	1	2	324
		% of marital status	67.01	33.33	100.00	
		% of willingness to vaccinate	99.07	0.31	0.62	
		% of the group	66.32	0.21	0.41	66.94
	**No**	N	158	2	0	160
		% of marital status	32.99	66.67	0.00	
		% of willingness to vaccinate	98.75	1.25	0.00	
		% of the group	32.64	0.41	0.00	33.06

	**Total**	N	479	3	2	484
		% of the entire group	98.97	0.62	0.41	100.00
**Chi² Pearson: 2.520067, df=2, p=0.28364, N=484**						

Table S58: Willingness to vaccinate amongst Indian students with respect to whether the respondents have children.

**Do you have children?**					
			**No**	**One child**	**In total**
	**Yes**	N	316	8	324
		% of having children	66.53	88.89	
		% of willingness to vaccinate	97.53	2.47	
		% of the group	65.29	1.65	66.94

**Would you like to be vaccinated?**	**No**	N	159	1	160
		% of having children	33.47	11.11	
		% of willingness to vaccinate	99.38	0.63	
		% of the group	32.85	0.21	33.06
	**Total**	N	475	9	484
		% of the entire group	98.14	1.86	100.00
**Chi^2 Pearson: 1.995998, df=1, p=0.15772, N=484**					

Table S59: Willingness to vaccinate amongst Indian students taking into account with whom the respondents lived during the pandemic.

**I live with:**

			Alone	Roommates	Parents	Partner or spouse	Partner or spouse and children	In total
**Would you like to be vaccinated?**	**Yes**	N	3	11	306	2	2	324
		% of living with	75.00	84.62	66.23	66.67	100.00	
		% of willingness to vaccinate	0.93	3.40	94.44	0.62	0.62	
		% of the group	0.62	2.27	63.22	0.41	0.41	66.94
	**No**	N	1	2	156	1	0	160
		% of living with	25.00	15.38	33.77	33.33	0.00	
		% of willingness to vaccinate	0.63	1.25	97.50	0.63	0.00	
		% of the group	0.21	0.41	32.23	0.21	0.00	33.06
	**Total**	N	4	13	462	3	2	484
		% of the entire group	0.83	2.69	95.45	0.62	0.41	100.00
**Chi² Pearson: 3.044733, df=4, p=0.55037, N=484**								

Table S60: Willingness to vaccinate amongst Indian students with respect to one's work during the pandemic.

			Are you currently working?				
			No, I do not work	I work mentally	I work physically	I run my own business	In total
Would you like to be vaccinated?	Yes	N	302	11	8	3	324
		% of currently working	67.11	68.75	66.67	50.00	
		% of willingness to vaccinate	93.21	3.40	2.47	0.93	
		% of the group	62.40	2.27	1.65	0.62	66.94
	No	N	148	5	4	3	160
		% of currently working	32.89	31.25	33.33	50.00	
		% of willingness to vaccinate	92.50	3.13	2.50	1.88	
		% of the group	30.58	1.03	0.83	0.62	33.06
	Total	N	450	16	12	6	484
		% of the entire group	92.98	3.31	2.48	1.24	100.00
Chi² Pearson: 0.8080879, df=3, p=0.84753, N=484							

Table S61: Willingness to vaccinate amongst Indian students with respect to the fact whether one's used psychological/psychiatric services before the pandemic outbreak.

Did you use psychological/psychiatric services before the pandemics broke out?							
			**No**	**Yes, psychological**	**Yes, psychiatric**	**Yes, psychological and psychiatric**	**In total**
**Would you like to be vaccinated?**	**Yes**	N	314	4	3	3	324
		% of using services	67.38	50.00	42.86	100.00	
		% of willingness to vaccinate	96.91	1.23	0.93	0.93	
		% of the group	64.88	0.83	0.62	0.62	66.94
	**No**	N	152	4	4	0	160
		% of using services	32.62	50.00	57.14	0.00	
		% of willingness to vaccinate	95.00	2.50	2.50	0.00	
		% of the group	31.40	0.83	0.83	0.00	33.06
	**Total**	N	466	8	7	3	484
		% of the entire group	96.28	1.65	1.45	0.62	100.00
**Chi^2 Pearson: 4.394792, df=3, p=0.22187, N=484**							

Table S62: Willingness to vaccinate amongst Indian students with respect to the fact whether one's used psychological/psychiatric services during the pandemic.

Did you use psychological/psychiatric services during the pandemics?									
**Would you like to be vaccinated?**			**I generally do not use the help of a psychologist/pychiatrist**	**I had to start using the services of a psychologist/psychiatrist because I feel worse**	**I continue to use the services with the same frequency as before the pandemic**	**I need more frequent visits because I feel worse**	**I have difficulties with contacting a psychiatrist/psychologist due to the epidemic**	**I go to visits less often due to the improvement of my well-being**	**In total**
	**Yes**	N	302	12	3	3	0	4	324
		% of using services	67.56	80.00	42.86	75.00	0.00	44.44	
		% of willingness to vaccinate	93.21	3.70	0.93	0.93	0.00	1.23	
		% of the group	62.40	2.48	0.62	0.62	0.00	0.83	66.94
	**No**	N	145	3	4	1	2	5	160
		% of using services	32.44	20.00	57.14	25.00	100.00	55.56	

		% of willingness to vaccinate	90.63	1.88	2.50	0.63	1.25	3.13	
		% of the group of	29.96	0.62	0.83	0.21	0.41	1.03	33.06
	**Total**	N	447	15	7	4	2	9	484
		% of the entire group	92.36	3.10	1.45	0.83	0.41	1.86	100.00
**Chi² Pearson: 9.293982, df=5, p=0.09790, N=484**									

Table S63: Willingness to vaccinate amongst Indian students with respect to the fact whether the students were taking any supplements during the pandemic.

		**Do you take any supplements during the pandemics?**				
**Would you like to be vaccinated?**		**Yes, I continue to take those which I was taken before the pandemics and those which I've started**	**Yes, I've started to take them during the pandemics**	**Yes, I continue to take those which I was taking before the pandemics**	**No**	**In total**

		during the pandemics				
	Yes	N	8	58	27	231	324
		% of taking supplements	53.33	70.73	64.29	67.74	
		% of willingness to vaccinate	2.47	17.90	8.33	71.30	
		% of the group	1.67	12.08	5.63	48.13	67.50
	No	N	7	24	15	110	156
		% of taking supplements	46.67	29.27	35.71	32.26	
		% of willingness to vaccinate	4.49	15.38	9.62	70.51	
		% of the group	1.46	5.00	3.13	22.92	32.50
	Total	N	15	82	42	341	480
		% of the entire group	3.13	17.08	8.75	71.04	100.00
**Chi² Pearson: 1.969553, df=3, p=0.57875, N=480**							

Table S64: Willingness to vaccinate amongst Indian students with respect to the fact whether the students are being vaccinated against influenza.

**Are you being vaccinated against influenza?**				
		**No**	**Yes**	**In total**

Would you like to be vaccinated?	Yes	N	177	147	324
		% of being vaccinated	59.20	79.46	
		% of willingness to vaccinate	54.63	45.37	
		% of the group	36.57	30.37	66.94
	No	N	122	38	160
		% of being vaccinated	40.80	20.54	
		% of willingness to vaccinate	76.25	23.75	
		% of the group	25.21	7.85	33.06
	Total	N	299	185	484
		% of the entire group	61.78	38.22	100.00
**Chi² Pearson: 21.20282, df=1, p=0.00000, N=484**					

Table S65: Willingness to vaccinate amongst Indian students with respect to being already vaccinated during the pandemic.

Are you vaccinated against COVID-19?					
**Would you like to be vaccinated?**			**Yes**	**No**	**In total**
	**Yes**	N	144	180	324
		% of being vaccinated	97.96	53.41	
		% of willingness to vaccinate	44.44	55.56	
	**No**	N	3	157	160
		% of being vaccinated	2.04	46.59	
		% of willingness to vaccinate	1.88	98.13	
	**TOTAL**	N	147	337	484
**Chi² Pearson: 91.78233, df=1, p=0.0000, N=484**					

Table S66: Willingness to vaccinate amongst students with respect to their gender.

Gender					
			**Female**	**Male**	**In total**
**Would you like to be vaccinated?**	**Yes**	N	171	48	219
		% of gender	93.44	94.12	
		% of willingness to vaccinate	78.08	21.92	
		% of the group	73.08	20.51	93.59
	**No**	N	12	3	15
		% of gender	6.56	5.88	
		% of willingness to vaccinate	80.00	20.00	
		% of the group	5.13	1.28	6.41
	**Total**	N	183	51	234
		% of the entire group	78.21	21.79	100.00
**Chi^2 Pearson: 0.0302929, df=1, p=0.86183, N=234**					

Table S67: Vaccinated Mexican students during the pandemic with respect to their gender.

Are you vaccinated against COVID-19?					
**Gender**			**Yes**	**No**	**In total**
	**Female**	N	34	149	183
		% of being vaccinated	72.34	79.68	
		% of gender	18.58	81.42	
	**Male**	N	13	38	51
		% of being vaccinated	27.66	20.32	
		% of gender	25.49	74.51	
	**TOTAL**		47	187	234
**Chi² Pearson: 1.186, df=1, p=0.275, N=234**					

Table S68: Willingness to vaccinate amongst Mexican students with respect to their field of study.

Field of study								
			**Medical studies**	**Social sciences**	**Technical science**	**Artistic or humanistic studies**	**Sciences**	**In total**
**Would you like to be vaccinated?**	**Yes**	N	139	41	15	13	11	219
		% of field of study	96.53	87.23	93.75	86.67	91.67	
		% of willingness to vaccinate	63.47	18.72	6.85	5.94	5.02	
		% of the group	59.40	17.52	6.41	5.56	4.70	93.59
	**No**	N	5	6	1	2	1	15
		% of field of study	3.47	12.77	6.25	13.33	8.33	
		% of willingness to vaccinate	33.33	40.00	6.67	13.33	6.67	
		% of the group	2.14	2.56	0.43	0.85	0.43	6.41
	**Total**	N	144	47	16	15	12	234
		% of the entire group	61.54	20.09	6.84	6.41	5.13	100.00
**Chi^2 Pearson: 6.509548, df=4, p=0.16419, N=234**								

Table S69: Vaccinated Mexican students with respect to their field of study during the pandemic.

Are you vaccinated against COVID-19?					
			**Yes**	**No**	**In total**
**Field of study**	**Medical studies**	N	31	113	144
		% of being vaccinated	65.96	60.43	
		% of field of study	21.53	78.47	
	**Social sciences**	N	8	39	47
		% of being vaccinated	17.02	20.86	
		% of field of study	17.02	82.98	
	**Technical science**	N	4	12	16
		% of being vaccinated	8.51	6.42	
		% of field of study	25.00	75.00	
	**Artistic or humanistic studies**	N	4	11	15
		% of being vaccinated	8.51	5.88	
		% of field of study	26.67	73.33	

	**Sciences**	N	0	12	12
		% of being vaccinated	0.00	6.42	
		% of field of study	0.00	100.00	
	**TOTAL**	N	47	187	234
**Chi² Pearson: 4.123, df=4, p=0.389, N=234**					

Table S70: Willingness to vaccinate amongst Mexican students with respect to their field of study during the pandemic.

**How do you currently study at your university (during this semester)?**								
**Would you like to be vaccinated?**			**Completely online**	**More than 50% classes online**	**About 30-50% classes online**	**Up to 30% classes online**	**Completely through direct contact**	**In total**
	**Yes**	N	196	9	6	2	6	219
		% of studying form	93.33	90.00	100.00	100.00	100.00	
		% of willingness to vaccinate	89.50	4.11	2.74	0.91	2.74	
		% of the group	83.76	3.85	2.56	0.85	2.56	93.59
	**No**	N	14	1	0	0	0	15

		% pf studying form	6.67	10.00	0.00	0.00	0.00	
		% of willingness to vaccinate	93.33	6.67	0.00	0.00	0.00	
		% of the group	5.98	0.43	0.00	0.00	0.00	6.41
	**Total**	N	210	10	6	2	6	234
		% of the entire group	89.74	4.27	2.56	0.85	2.56	100.00
**Chi² Pearson: 1.196712, df=4, p=0.87864, N=234**								

Table S71: Vaccinated Mexican students with respect to the current types of classes/studying type at university during the pandemic.

**Are you vaccinated against COVID-19?**					
			**Yes**	**No**	**In total**
	**Completely online**	N	37	173	210
		% of being vaccinated	78.72	92.51	

		% of studying form	17.62	82.38	
Way of conducting classes at the university	More than 50% classes online	N	4	6	10
		% of being vaccinated	8.51	3.21	
		% of studying form	40.00	60.00	
	About 30-50% classes online	N	1	5	6
		% of being vaccinated	2.13	2.67	
		% of studying form	16.67	83.33	
	Up to 30% classes online	N	2	0	2
		% of being vaccinated	4.26	0.00	
		% of studying form	100.00	0.00	
	Completely through direct contact	N	3	3	6
		% of being vaccinated	6.38	1.60	
		% of studying form	50.00	50.00	
	TOTAL	N	47	187	234
**Chi² Pearson: 14.612, df=4, p=0.005, N= 234**					

Table S72: Willingness to vaccinate amongst Mexican students with respect to their place of residence during the pandemic.

**Place of residence**

			A city with over 600 thous residents	A city between 100 and 600 thous residents	A city between 20 and 100 thous residents	The town below 20 thous residents	Village	In total
**Would you like to be vaccinated?**	**Yes**	N	123	51	27	13	5	219
		% of place of residence	94.62	96.23	96.43	86.67	62.50	
		% of willingness to vaccinate	56.16	23.29	12.33	5.94	2.28	
		% of the group	52.56	21.79	11.54	5.56	2.14	93.59
	**No**	N	7	2	1	2	3	15
		% of place of residence	5.38	3.77	3.57	13.33	37.50	
		% of willingness to vaccinate	46.67	13.33	6.67	13.33	20.00	
		% of the group	2.99	0.85	0.43	0.85	1.28	6.41
	**Total**	N	130	53	28	15	8	234
		% of the entire group	55.56	22.65	11.97	6.41	3.42	100.00
**Chi^2 Pearson: 15.30563, df=4, p=0.0041, N=234**								

Table S73: Willingness to vaccinate amongst Mexican students with respect to their marital status during the pandemic.

			Marital status				
			**Single**	**Informal relationship**	**Married**	**Divorcee**	**In total**
**Would you like to be vaccinated?**	**Yes**	N	186	26	5	2	219
		% of marital status	94.90	89.66	71.43	100.00	
		% of willingness to vaccinate	84.93	11.87	2.28	0.91	
		% of the group	79.49	11.11	2.14	0.85	93.59
	**No**	N	10	3	2	0	15
		% of marital status	5.10	10.34	28.57	0.00	
		% of willingness to vaccinate	66.67	20.00	13.33	0.00	
		% of the group	4.27	1.28	0.85	0.00	6.41
	**Total**	N	196	29	7	2	234
		% of the entire group	83.76	12.39	2.99	0.85	100.00
**Chi² Pearson: 7.174770, df=3, p=0.06653, N=234**							

Table S74: Willingness to vaccinate amongst Mexican students with respect to whether the respondents have children.

			Do you have children?					
			**No**	**I am pregnant or the wife/partner is pregnant**	**One child**	**Two children**	**More than two children**	**In total**
**Would you like to be vaccinated?**	**Yes**	N	209	1	4	3	2	219
		% of having children	93.72	100.00	80.00	100.00	100.00	
		% of willingness to vaccinate	95.43	0.46	1.83	1.37	0.91	
		% of the group	89.32	0.43	1.71	1.28	0.85	93.59
	**No**	N	14	0	1	0	0	15
		% of having children	6.28	0.00	20.00	0.00	0.00	
		% of willingness to vaccinate	93.33	0.00	6.67	0.00	0.00	
		% of the group	5.98	0.00	0.43	0.00	0.00	6.41
	**Total**	N	223	1	5	3	2	234
		% of the entire group	95.30	0.43	2.14	1.28	0.85	100.00
**Chi^2 Pearson: 1.956636, df=4, p=0.74373, N=234**								

Table S75: Willingness to vaccinate amongst Mexican students taking into account with whom the respondents lived during the pandemic.

I live with:								
Would you like to be vaccinated?			**Alone**	**Roommates**	**Parents**	**Partner or spouse**	**Partner or spouse and children**	**In total**
	**Yes**	N	11	11	190	4	3	219
		% of living with	100.00	100.00	94.06	57.14	100.00	
		% of willingness to vaccinate	5.02	5.02	86.76	1.83	1.37	
		% of the group	4.70	4.70	81.20	1.71	1.28	93.59
	**No**	N	0	0	12	3	0	15
		% of living with	0.00	0.00	5.94	42.86	0.00	
		% of willingness to vaccinate	0.00	0.00	80.00	20.00	0.00	
		% of the group	0.00	0.00	5.13	1.28	0.00	6.41
	**Total**	N	11	11	202	7	3	234
		% of the entire group	4.70	4.70	86.32	2.99	1.28	100.00
**Chi² Pearson: 17.28601, df=4, p=0.00170, N=234**								

Table S76: Willingness to vaccinate amongst Mexican students with respect to one's work during the pandemic.

Are you currently working?							
			**No, I do not work**	**I work mentally**	**I work physically**	**I run my own business**	**In total**
**Would you like to be vaccinated?**	**Yes**	N	160	26	23	10	219
		% of currently working	94.67	92.86	100.00	71.43	
		% of willingness to vaccinate	73.06	11.87	10.50	4.57	
		% of the group	68.38	11.11	9.83	4.27	93.59
	**No**	N	9	2	0	4	15
		% of currently working	5.33	7.14	0.00	28.57	
		% of willingness to vaccinate	60.00	13.33	0.00	26.67	

		% of the group	3.85	0.85	0.00	1.71	6.41
	**Total**	N	169	28	23	14	234
		% of the entire group	72.22	11.97	9.83	5.98	100.00
**Chi² Pearson: 13.39256, df=3, p=0.0038, N=234**							

Table S77: Willingness to vaccinate amongst Mexican students with respect to the fact whether one's used psychological/psychiatric services before the pandemic outbreak.

**Did you use psychological/psychiatric services before the pandemics broke out?**							
			**No**	**Yes, psychological**	**Yes, psychiatric**	**Yes, psychological and psychiatric**	**In total**
	**Yes**	N	130	69	4	16	219
		% of using services	92.86	94.52	100.00	94.12	

**Would you like to be vaccinated?**		% of willingness to vaccinate	59.36	31.51	1.83	7.31	
		% of the group	55.56	29.49	1.71	6.84	93.59
	**No**	N	10	4	0	1	15
		% of using services	7.14	5.48	0.00	5.88	
		% of willingness to vaccinate	66.67	26.67	0.00	6.67	
		% of the group	4.27	1.71	0.00	0.43	6.41
	**Total**	N	140	73	4	17	234
		% of the entire group	59.83	31.20	1.71	7.26	100.00
**Chi² Pearson: 0.5125372, df=3, p=0.9161, N=234**							

Table S78: Willingness to vaccinate amongst Mexican students with respect to the fact whether one's used psychological/psychiatric services during the pandemic.

**Did you use psychological/psychiatric services during the pandemics?**

Would you like to be vaccinated?			I generally do not use the help of a psychologist/psychiatrist	I had to start using the services of a psychologist/psychiatrist because I feel worse	I continue to use the services with the same frequency as before the pandemic	I need more frequent visits because I feel worse	I have difficulties with contacting a psychiatrist/psychologist due to the epidemic	I go to visits less often due to the improvement of my well-being	In total
	Yes	N	121	36	21	8	29	4	219
		% of using services	92.37	94.74	100.00	100.00	93.55	80.00	
		% of willingness to vaccinate	55.25	16.44	9.59	3.65	13.24	1.83	
		% of the group	51.71	15.38	8.97	3.42	12.39	1.71	93.59
	No	N	10	2	0	0	2	1	15
		% of using services	7.63	5.26	0.00	0.00	6.45	20.00	
		% of willingness to vaccinate	66.67	13.33	0.00	0.00	13.33	6.67	
		% of the group	4.27	0.85	0.00	0.00	0.85	0.43	6.41
	Total	N	131	38	21	8	31	5	234
		% of the entire group	55.98	16.24	8.97	3.42	13.25	2.14	100.00
**Chi² Pearson: 3.935694, df=5, p=0.55871, N=234**									

Table S79: Willingness to vaccinate amongst Mexican students with respect to the fact whether the students were taking any supplements during the pandemic.

			Do you take any supplements during the pandemics?				
**Would you like to be vaccinated?**			**Yes, I continue to take those which I was taken before the pandemics and those which I've started during the pandemics**	**Yes, I've started to take them during the pandemics**	**Yes, I continue to take those which I was taking before the pandemics**	**No**	**In total**
	**Yes**	N	13	58	41	106	218
		% of taking suplements	76.47	95.08	95.35	94.64	
		% of willingness to vaccinate	5.96	26.61	18.81	48.62	
		% of the group	5.58	24.89	17.60	45.49	93.56
	**No**	N	4	3	2	6	15
		% of taking supplements	23.53	4.92	4.65	5.36	
		% of willingness to vaccinate	26.67	20.00	13.33	40.00	
		% of the group	1.72	1.29	0.86	2.58	6.44
	**Total**	N	17	61	43	112	233
		% of the entire group	7.30	26.18	18.45	48.07	100.00
**Chi² Pearson: 8.923717, df=3, p=0.03032, N=233**							

Table S80: Willingness to vaccinate amongst Mexican students with respect to the fact whether the students are being vaccinated against influenza.

			**Are you being vaccinated against influenza?**		
			**No**	**Yes**	**In total**
**Would you like to be vaccinated?**	**Yes**	N	41	178	219
		% of being vaccinated	80.39	97.27	
		% of willingness to vaccinate	18.72	81.28	
		% of the group	17.52	76.07	93.59
	**No**	N	10	5	15
		% of being vaccinated	19.61	2.73	
		% of willingness to vaccinate	66.67	33.33	
		% of the group	4.27	2.14	6.41
	**Total**	N	51	183	234
		% of the entire group	21.79	78.21	100.00
**Chi² Pearson: 18.93304, df=1, p=0.00001, N=234**					

Table S81: Willingness to vaccinate amongst Mexico students with respect to being already vaccinated during the pandemic.

Are you vaccinated against COVID-19?					
**Would you like to be vaccinated?**			**Yes**	**No**	**In total**
	**Yes**	N	47	172	219
		% of being vaccinted	100.00	91.98	
		% of willingness to vaccinate	21.46	78.54	
	**No**	N	0	15	15
		% of being vaccinated	0.00	8.02	
		% of willingness to vaccinte	0.00	100.00	
	**TOTAL**	N	47	187	234
**Chi² Pearson: 4.0282, df=1, p=0.044, N=234**					

Table S82: Willingness to vaccinate amongst Egyptian students with respect to their gender.

Gender					
			**Female**	**Male**	**In total**
**Would you like to be vaccinated?**	**Yes**	N	177	166	343
		% of gender	53.47	70.64	
		% of willingness to vaccinate	51.60	48.40	
		% of the group	31.27	29.33	60.60
	**No**	N	154	69	223
		% of gender	46.53	29.36	
		% of willingness to	69.06	30.94	

		vaccinate			
		% of the group	27.21	12.19	39.40
	**Total**	N	331	235	566
		% of the entire group	58.48	41.52	100.00
**Chi² Pearson: 16.95701, df=1, p=0.00004, N=566**					

Table S83: Vaccinated Egyptian students during the pandemic with respect to their gender.

**Are you vaccinated against COVID-19?**					
			**Yes**	**No**	**In total**
	**Female**	N	50	281	331
		% of being vaccinated	61.73	57.94	

**Gender**		% of gender	15.11	84.89	
	**Male**	N	31	204	235
		% of being vaccinated	38.27	42.06	
		% of gender	13.19	86.81	
	**TOTAL**	N	81	485	566
**Chi^2 Pearson: 0.410, df=1, p=0.521, N=566**					

Table S84: Willingness to vaccinate amongst Egyptian students with respect to their field of study during the pandemic.

Field of study								
			**Medical studies**	**Social sciences**	**Technical science**	**Artistic or humanistic studies**	**Sciences**	**In total**
**Would you like to be vaccinated?**	**Yes**	N	189	52	40	39	23	343
		% of field of study	64.51	63.41	52.63	57.35	48.94	
		% of willingness to vaccinate	55.10	15.16	11.66	11.37	6.71	
		% of the group	33.39	9.19	7.07	6.89	4.06	60.60
	**No**	N	104	30	36	29	24	223
		% of field of study	35.49	36.59	47.37	42.65	51.06	
		% of willingness to vaccinate	46.64	13.45	16.14	13.00	10.76	
		% of the group	18.37	5.30	6.36%	5.12	4.24	39.40
	**Total**	N	293	82	76	68	47	566
		% of the entire group	51.77	14.49	13.43	12.01	8.30	100.00
**Chi^2 Pearson: 7.142911, df=4, p=0.12853,N=566**								

Table S85: Vaccinated Egyptian students with respect to their field of study during the pandemic.

Are you vaccinated against COVID-19?					
			**Yes**	**No**	**In total**
**Field of study**	**Medical studies**	N	43	250	293
		% of being vaccinated	53.09	51.55	
		% of field of study	14.68	85.32	
	**Social sciences**	N	17	65	82
		% of being vaccinated	20.99	13.40	
		% of field of study	20.73	79.27	
	**Technical science**	N	7	69	76
		% of being vaccinated	8.64	14.23	
		% of field of study	9.21	90.79	
	**Artistic or humanistic studies**	N	6	62	68
		% of being vaccinated	7.41	12.78	
		% of field of study	8.82	91.18	
	**Sciences**	N	8	39	47
		% of being vaccinted	9.88	8.04	
		% of field of study	17.02	82.98	
	**TOTAL**	N	81	485	566
**Chi^2 Pearson: 6.352, df=4, p=0.174, N=566**					

Table S86: Willingness to vaccinate amongst Egyptian students with respect to the current types of classes/studying type at university during the pandemic.

How do you currently study at your university (during this semester)?							
			**Completely online**	**More than 50% classes online**	**About 30-50% classes online**	**Up to 30% classes online**	**In total**
**Would you like to be vaccinated?**	**Yes**	N	28	138	103	74	343
		% of studying form	52.83	65.71	57.54	59.68	
		% of willingness to vaccinate	8.16	40.23	30.03	21.57	
		% of the group	4.95	24.38	18.20	13.07	60.60
	**No**	N	25	72	76	50	223
		% of field of study	47.17	34.29	42.46	40.32	
		% of willingness to vaccinate	11.21	32.29	34.08	22.42	
		% of the group	4.42	12.72	13.43	8.83	39.40
	**Total**	N	53	210	179	124	566
		% of the entire group	9.36	37.10	31.63	21.91	100.00
**Chi^2 Pearson: 4.385906, df=3, p=0.22270, N=566**							

Table S87: Vaccinated Egyptian students with respect to the current types of classes/studying type at university during the pandemic.

**Are you vaccinated against COVID-19?**					
**Way of conducting classes at the university**			**Yes**	**No**	**In total**
	**Completely online**	N	7	46	53
		% of being vaccinated	8.64	9.48	
		% of studying form	13.21	86.79	
	**More than 50% classes online**	N	29	181	210
		% of being vaccinated	35.80	37.32	
		% of studying form	13.81	86.19	
	**About 30-50% classes online**	N	23	156	179
		% of being vaccinated	28.40	32.16	
		% of studying form	12.85	87.15	
	**Up to 30% classes online**	N	22	102	124
		% of being vaccinated	27.16	21.03	
		% of studying form	17.74	82.26	
	**TOTAL**	N	81	485	566
**Chi^2 Pearson: 1.597, df=3, p=0.659, N= 566**					

Table S88: Willingness to vaccinate amongst Egyptian students with respect to their place of residence during the pandemic.

Place of residence							
			**A city with over 600 thous residents**	**A city between 100 and 600 thous residents**	**A city between 20 and 100 thous residents**	**Village**	**In total**
**Would you like to be vaccinated?**	**Yes**	N	119	8	6	210	343
		% of place of residence	57.49	44.44	66.67	63.25	
		% of willingness to vaccinate	34.69	2.33	1.75	61.22	
		% of the group	21.02	1.41	1.06	37.10	60.60
	**No**	N	88	10	3	122	223
		% of place of residence	42.51	55.56	33.33	36.75	
		% of willingness to vaccinate	39.46	4.48	1.35	54.71	
		% of the group	15.55	1.77	0.53	21.55	39.40
	**Total**	N	207	18	9	332	566
		% of the entire group	36.57	3.18	1.59	58.66	100.00

Chi² Pearson: 3.924757, df=3, p=0.26970, N=566

Table S89: Willingness to vaccinate amongst Egyptian students with respect to their marital status during the pandemic.

Marital status					
			**Single**	**Married**	**In total**
**Would you like to be vaccinated?**	**Yes**	N	321	22	343
		% of marital status	59.44	84.62	
		% of willingness to vaccinate	93.59	6.41	
		% of the group	56.71	3.89	60.60
	**No**	N	219	4	223
		% of marital status	40.56	15.38	
		% of willingness to vaccinate	98.21	1.79	

		% of the group	38.69	0.71	39.40
	**Total**	N	540	26	566
		% of the entire group	95.41	4.59	100.00
**Chi² Pearson: 6.582387, df=1, p=0.01030, N=566**					

Table S90: Willingness to vaccinate amongst Egyptian students with respect to whether the respondents have children.

**Do you have children?**						
		**No**	**I am pregnant or the wife/partner is pregnant**	**One child**	**In total**

**Would you like to be vaccinated?**	**Yes**	N	330	6	7	343
		% of having children	60.11	85.71	70.00	
		% of willingness to vaccinate	96.21	1.75	2.04	
		% of the group	58.30	1.06	1.24	60.60
	**No**	N	219	1	3	223
		% of having children	39.89	14.29	30.00	
		% of willingness to vaccinate	98.21	0.45	1.35	
		% of the group	38.69	0.18	0.53	39.40
	**Total**	N	549	7	10	566
		% of the entire group	97.00	1.24	1.77	100.00
**Chi^2 Pearson: 2.274599, df=2, p=0.32068, N=566**						

Table S91: Willingness to vaccinate amongst Egyptian students taking into account with whom the respondents lived during the pandemic.

			I live with:				
**Would you like to be vaccinated?**			**Roommates**	**Parents**	**Partner or spouse**	**Partner or spouse and children**	**In total**
	**Yes**	N	41	284	11	7	343
		% of living with	54.67	60.55	91.67	70.00	
		% of willingness to vaccinate	11.95	82.80	3.21	2.04	
		% of the group	7.24	50.18	1.94	1.24	60.60
	**No**	N	34	185	1	3	223
		% of living with	45.33	39.45	8.33	30.00	
		% of willingness to vaccinate	15.25	82.96	0.45	1.35	
		% of the group	6.01	32.69	0.18	0.53	39.40
	**Total**	N	75	469	12	10	566
		% of the entire group	13.25	82.86	2.12	1.77	100.00
**Chi² Pearson: 6.327025, df=3, p=0.09674, N=566**							

Table S92: Willingness to vaccinate amongst Egyptian students with respect to one's work during the pandemic.

Are you currently working?							
			**No, I do not work**	**I work mentally**	**I work physically**	**I run my own business**	**In total**
**Would you like to be vaccinated?**	**Yes**	N	294	30	17	2	343
		% of currently working	61.38	66.67	42.50	100.00	
		% of willingness to vaccinate	85.71	8.75	4.96	0.58	
		% of the group	51.94	5.30	3.00	0.35	60.60
	**No**	N	185	15	23	0	223
		% of currently working	38.62	33.33	57.50	0.00	
		% of willingngess to vaccinate	82.96	6.73	10.31	0.00	
		% of the group	32.69	2.65	4.06	0.00	39.40
	**Total**	N	479	45	40	2	566
		% of the entire group	84.63	7.95	7.07	0.35	100.00
**Chi^2 Pearson: 7.603855, df=3, p=0.05495, N=566**							

Table S93: Willingness to vaccinate amongst Egyptian students with respect to the fact whether one's used psychological/psychiatric services before the pandemic outbreak.

Did you use psychological/psychiatric services before the pandemics broke out?						
			**No**	**Yes, psychological**	**Yes, psychiatric**	**In total**
**Would you like to be vaccinated?**	**Yes**	N	335	5	3	343
		% of using services	61.13	38.46	60.00	
		% of willingness to vaccinate	97.67	1.46	0.87	
		% of the group	59.19	0.88	0.53	60.60
	**No**	N	213	8	2	223
		% of using services	38.87	61.54	40.00	
		% of willingness to vaccinate	95.52	3.59	0.90	
		% of the group	37.63	1.41	0.35	39.40
	**Total**	N	548	13	5	566
		% of the entire group	96.82	2.30	0.88	100.00
**Chi² Pearson: 2.734093, df=2, p=0.25486, N=566**						

Table S94: Willingness to vaccinate amongst Egyptian students with respect to the fact whether one's used psychological/psychiatric services during the pandemic.

			Did you use psychological/psychiatric services during the pandemics?					
**Would you like to be vaccinated?**			**I generally do not use the help of a psychologist/psychiatrist**	**I had to start using the services of a psychologist/psychiatrist because I feel worse**	**I continue to use the services with the same frequency as before the pandemic**	**I need more frequent visits because I feel worse**	**I have difficulties with contacting a psychiatrist/psychologist due to the epidemic**	**In total**
	**Yes**	N	335	0	4	2	2	343
		% of using services	61.13	0.00	50.00	40.00	66.67	
		% of willingness to vaccinate	97.67	0.00	1.17	0.58	0.58	
		% of the group	59.19	0.00	0.71	0.35	0.35	60.60
	**No**	N	213	2	4	3	1	223

		% of using services	38.87	100.00	50.00	60.00	33.33	
		% of willingness to vaccinate	95.52	0.90	1.79	1.35	0.45	
		% of the group	37.63	0.35	0.71	0.53	0.18	39.40
	**Total**	N	548	2	8	5	3	566
		% of the entire group	96.82	0.35	1.41	0.88	0.53	100.00
**Chi² Pearson: 4.452354, df=4, p=0.34823, N=566**								

Table S95: Willingness to vaccinate amongst Egyptian students with respect to the fact whether the students were taking any supplements during the pandemic.

**Do you take any supplements during the pandemics?**						
**Would you like to be vaccinated?**		**Yes, I continue to take those which I was taken before the pandemics and those which I've started**	**Yes, I've started to take them during the pandemics**	**Yes, I continue to take those which I was taking before the pandemics**	**No**	**In total**

			during the pandemics				
	Yes	N	9	50	25	259	343
		% of taking suplements	42.86	57.47	48.08	63.79	
		% of willingngess to vaccinate	2.62	14.58	7.29	75.51	
		% of the group	1.59	8.83	4.42	45.76	60.60
	No	N	12	37	27	147	223
		% of taking supplements	57.14	42.53	51.92	36.21	
		% of willingness to vaccinate	5.38	16.59	12.11	65.92	
		% of the group	2.12	6.54	4.77	25.97	39.40
	Total	N	21	87	52	406	566
		% of the entire group	3.71	15.37	9.19	71.73	100.00
**Chi² Pearson: 8.274833, df=3, p=0.04066, N=566**							

Table S96: Willingness to vaccinate amongst Egyptian students with respect to the fact whether the students are being vaccinated against influenza.

**Are you being vaccinated against influenza?**			
		**No**	**In total**

**Would you like to be vaccinated?**	**Yes**	N	343	343
		% of being vaccinated	60.60	
		% of willingness to vaccinate	100.00	
		% of the group	60.60	60.60
	**No**	N	223	223
		% of being vaccinated	39.40	
		% of willingness to vaccinate	100.00	
		% of the group	39.40	39.40
	**Total**	N	566	566
		% of the entire group	100.00	100.00
**Chi² Pearson: 0.000000. df=0. p=1.0000. N=566**				

Table S97: Willingness to vaccinate amongst Egyptian students with respect to being already vaccinated during the pandemic.

Are you vaccinated against COVID-19?					
			**Yes**	**No**	**In total**
**Would you like to be vaccinated?**	**Yes**	N	81	262	343
		% of being vaccinated	100.00	54.02	
		% of willingness to vaccinate	23.62	76.38	
	**No**	N	0	223	223
		% of being vaccinated	0.00	45.98	
		% of willingness to vaccinte	0.00	100.00	
	**TOTAL**	N	81	485	566
**Chi² Pearson: 61.456, df=1, p=0.0000, N=566**					

Table S98: Willingness to vaccinate amongst Filipino students with respect to their gender.

Gender					
			**Female**	**Male**	**In total**
**Would you like to be vaccinated?**	**Yes**	N	1192	537	1729
		% of gender	84.24	81.24	
		% of willingness to vaccinate	68.94	31.06	
		% of the group	57.42	25.87	83.29
	**No**	N	223	124	347
		% of gender	15.76	18.76	
		% of willingness to vaccinate	64.27	35.73	
		% of the group	10.74	5.97	16.71
	**Total**	N	1415	661	2076
		% of the entire group	68.16	31.84	100.00
**Chi^2 Pearson: 2.912242, df=1, p=0.0879, N=2076**					

Table S99: Vaccinated Filipino students during the pandemic with respect to their gender.

Are you vaccinated against COVID-19?					
Gender			Yes	No	In total
	Female	N	182	1233	1415
		% of being vaccinated	67.16	68.31	
		% of gender	12.86	87.14	
	Male	N	89	572	661
		% of being vaccinated	32.84	31.69	
		% of gender	13.46	86.54	
	TOTAL	N	271	1805	2076
Chi² Pearson: 0.143, df=1, p=0.704, N=2076					

Table S100: Willingness to vaccinate amongst Filipino students with respect to their field of study during the pandemic.

Field of study								
			**Medical studies**	**Social sciences**	**Technical science**	**Artistic or humanistic studies**	**Sciences**	**In total**
**Would you like to be vaccinated?**	**Yes**	N	305	554	394	192	284	1729
		% of field of study	73.49	85.89	88.34	82.40	84.27	
		% of willingness to vaccinate	17.64	32.04	22.79	11.10	16.43	
		% of the group	14.69	26.69	18.98	9.25	13.68	83.29
	**No**	N	110	91	52	41	53	347
		% of field of study	26.51	14.11	11.66	17.60	15.73	
		% of willingness to vaccinate	31.70	26.22	14.99	11.82	15.27	
		% of the group	5.30	4.38	2.50	1.97	2.55	16.71
	**Total**	N	415	645	446	233	337	2076
		% of the entire group	19.99	31.07	21.48	11.22	16.23	100.00
**Chi^2 Pearson: 40.28157, df=4, p=0.00000, N=2076**								

Table S101: Vaccinated Filipino students with respect to their field of study during the pandemic.

Are you vaccinated against COVID-19?					
Field of study			**Yes**	**No**	**In total**
	**Medical studies**	N	132	283	415
		% of being vaccinated	48.71	15.68	
		% of field of study	31.81	68.19	
	**Social sciences**	N	66	579	645
		% of being vaccinated	24.35	32.08	
		% of field of study	10.23	89.77	
	**Technical science**	N	33	413	446
		% of being vaccinated	12.18	22.88	
		% of field of study	7.40	92.60	
		N	21	212	233
		% of being vaccinated	7.75	11.75	

	**Artistic or humanistic studies**	% of field of study	9.01	90.99	
	**Sciences**	N	19	318	337
		% of being vaccinated	7.01	17.62	
		% of field of study	5.64	94.36	
	**TOTAL**	N	271	1805	2076
**Chi² Pearson: 165.362, df=4, p=0.0000, N=2076**					

Table S102: Willingness to vaccinate amongst Filipino students with respect to the current types of classes/studying type at university during the pandemic.

**How do you currently study at your university (during this semester)?**								
			**Completely online**	**More than 50% classes online**	**About 30-50% classes online**	**Up to 30% classes online**	**Completely through direct contact**	**In total**
**Would you like to be vaccinated?**	**Yes**	N	1502	95	65	37	30	1729
		% of studying form	82.08	95.00	86.67	94.87	93.75	
		% of willingness to vaccinate	86.87	5.49	3.76	2.14	1.74	

		% of the group	72.35	4.58	3.13	1.78	1.45	83.29
	**No**	N	328	5	10	2	2	347
		% of studying form	17.92	5.00	13.33	5.13	6.25	
		% of willingness to vaccinate	94.52	1.44	2.88	0.58	0.58	
		% of the group	15.80	0.24	0.48	0.10	0.10	16.71
	**Total**	N	1830	100	75	39	32	2076
		% of the entire group	88.15	4.82	3.61	1.88	1.54	100.00
**Chi² Pearson: 18.67316, df=4, p=0.00091, N=2076**								

Table S103: Vaccinated Filipino students with respect to the current types of classes/studying type at university during the pandemic.

**Are you vaccinated against COVID-19?**				
		**Yes**	**No**	**In total**

**Way of conducting classes at the university**	**Completely online**	N	245	1585	1830
		% of being vaccinated	90.41	87.81	
		% of studying form	13.39	86.61	
	**More than 50% classes online**	N	10	90	100
		% of being vaccinated	3.69	4.99	
		% of studying form	10.00	90.00	
	**About 30-50% classes online**	N	9	66	75
		% of being vaccinated	3.32	3.66	
		% of studying form	12.00	88.00	
	**Up to 30% classes online**	N	3	36	39
		% of being vaccinated	1.11	1.99	
		% of studying form	7.69	92.31	
	**Completely through direct contact**	N	4	28	32
		% of being vaccinated	1.48	1.55	
		% of studying form	12.50	87.50	
	**TOTAL**	N	271	1805	2076
**Chi^2 Pearson: 2.071, df=4, p=0.722, N= 2076**					

Table S104: Willingness to vaccinate amongst Filipino students with respect to their place of residence during the pandemic.

Place of residence								
			**A city with over 600 thous residents**	**A city between 100 and 600 thous residents**	**A city between 20 and 100 thous residents**	**The town below 20 thous residents**	**Village**	**In total**
**Would you like to be vaccinated?**	**Yes**	N	477	448	330	179	295	1729
		% of place of residence	80.71	84.69	85.27	87.32	81.04	
		% of willingness to vaccinate	27.59	25.91	19.09	10.35	17.06	
		% of the group	22.98	21.58	15.90	8.62	14.21	83.29
	**No**	N	114	81	57	26	69	347
		% of place of residence	19.29	15.31	14.73	12.68	18.96	
		% of willingness to vaccinate	32.85	23.34	16.43	7.49	19.88	
		% of the group	5.49	3.90	2.75	1.25	3.32	16.71
	**Total**	N	591	529	387	205	364	2076
		% of the entire group	28.47	25.48	18.64	9.87	17.53	100.00
**Chi² Pearson: 8.365739, df=4, p=0.07906, N=2076**								

Table S105: Willingness to vaccinate amongst Filipino students with respect to their marital status during the pandemic.

Marital status						
			Single	Informal relationship	Married	In total
Would you like to be vaccinated?	Yes	N	1654	22	53	1729
		% of marital status	83.24	84.62	84.13	
		% of willingness to vaccinate	95.66	1.27	3.07	
		% of the group	79.67	1.06	2.55	83.29
	No	N	333	4	10	347
		% of marital status	16.76	15.38	15.87	
		% of willingness to vaccinate	95.97	1.15	2.88	
		% of the group	16.04	0.19	0.48	16.71
	Total	N	1987	26	63	2076
		% of the entire group	95.71	1.25	3.03	100.00
Chi^2 Pearson:0.0678947, df=2, p=0.96662, N=2076						

Table S106: Willingness to vaccinate amongst Filipino students with respect to whether the respondents have children.

			Do you have children?					
			**No**	**I am pregnant or the wife/partner is pregnant**	**One child**	**Two children**	**More than two children**	**In total**
**Would you like to be vaccinated?**	**Yes**	N	1662	3	44	16	4	1729
		% of having children	83.10	75.00	93.62	76.19	100.00	
		% of willingness to vaccinate	96.12	0.17	2.54	0.93	0.23	
		% of the group	80.06	0.14	2.12	0.77	0.19	83.29
	**No**	N	338	1	3	5	0	347
		% of having children	16.90	25.00	6.38	23.81	0.00	
		% of willingness to vaccinate	97.41	0.29	0.86	1.44	0.00	
		% of the group	16.28	0.05	0.14	0.24	0.00	16.71
	**Total**	N	2000	4	47	21	4	2076
		% of the entire group	96.34	0.19	2.26	1.01	0.19	100.00
**Chi² Pearson: 5.412578, df=4, p=0.24752, N=2076**								

Table S107: Willingness to vaccinate amongst Filipino students taking into account with whom the respondents lived during the pandemic.

			I live with:						
			**Alone**	**Roommates**	**Parents**	**Partner or spouse**	**Partner or spouse and children**	**Children**	**In total**
**Would you like to be vaccinated?**	**Yes**	N	77	68	1484	46	47	7	1729
		% of living with	85.56	91.89	82.77	79.31	87.04	100.00	
		% of willingness to vaccinate	4.45	3.93	85.83	2.66	2.72	0.40	
		% of the group	3.71	3.28	71.48	2.22	2.26	0.34	83.29
	**No**	N	13	6	309	12	7	0	347
		% of living with	14.44	8.11	17.23	20.69	12.96	0.00	
		% of willingness to vaccinate	3.75	1.73	89.05	3.46	2.02	0.00	
		% of the group	0.63	0.29	14.88	0.58	0.34	0.00	16.71
	**Total**	N	90	74	1793	58	54	7	2076
		% of the entire group	4.34	3.56	86.37	2.79	2.60	0.34	100.00
**Chi² Pearson: 7.226791, df=5, p=0.20431, N=2076**									

Table S108: Willingness to vaccinate amongst Filipino students with respect to one's work during the pandemic.

**Are you currently working?**							
**Would you like to be vaccinated?**			**No, I do not work**	**I work mentally**	**I work physically**	**I run my own business**	**In total**
	**Yes**	N	1232	200	214	83	1729
		% of currently working	83.75	78.43	84.92	84.69	
		% of willingness to vaccinate	71.26	11.57	12.38	4.80	
		% of the group	59.34	9.63	10.31	4.00	83.29
	**No**	N	239	55	38	15	347
		% of currently working	16.25	21.57	15.08	15.31	

		% of willingness to vaccinate	68.88	15.85	10.95	4.32	
		% of the group	11.51	2.65	1.83	0.72	16.71
	**Total**	N	1471	255	252	98	2076
		% of the entire group	70.86	12.28	12.14	4.72	100.00
**Chi² Pearson: 5.170238, df=3, p=0.15975, N=2076**							

Table S109: Willingness to vaccinate amongst Filipino students with respect to the fact whether one's used psychological/psychiatric services before the pandemic outbreak.

**Did you use psychological/psychiatric services before the pandemics broke out?**						
		**No**	**Yes, psychological**	**Yes, psychiatric**	**Yes, psychological and psychiatric**	**In total**

**Would you like to be vaccinated?**	**Yes**	N	1537	98	55	39	1729
		% of using services	84.50	79.03	71.43	69.64	
		% of willingness to vaccinate	88.90	5.67	3.18	2.26	
		% of the group	74.04	4.72	2.65	1.88	83.29
	**No**	N	282	26	22	17	347
		% of using services	15.50	20.97	28.57	30.36	
		% of willingness to vaccinate	81.27	7.49	6.34	4.90	
		% of the group	13.58	1.25	1.06	0.82	16.71
	**Total**	N	1819	124	77	56	2076
		% of the entire group	87.62	5.97	3.71	2.70	100.00
**Chi^2 Pearson: 18.79240, df=3, p=0.00030, N=2076**							

Table S110: Willingness to vaccinate amongst Filipino students with respect to the fact whether one's used psychological/psychiatric services during the pandemic.

Did you use psychological/psychiatric services during the pandemics?									
**Would you like to be vaccinated?**			**I generally do not use the help of a psychologist/psychiatrist**	**I had to start using the services of a psychologist/psychiatrist because I feel worse**	**I continue to use the services with the same frequency as before the pandemic**	**I need more frequent visits because I feel worse**	**I have difficulties with contacting a psychiatrist/psychologist due to the epidemic**	**I go to visits less often due to the improvement of my well-being**	**In total**
	**Yes**	N	1365	123	21	26	174	20	1729
		% of using services	84.00	78.85	67.74	81.25	85.71	68.97	
		% of willingness to vaccinate	78.95	7.11	1.21	1.50	10.06	1.16	
		% of the group	65.75	5.92	1.01	1.25	8.38	0.96	83.29
	**No**	N	260	33	10	6	29	9	347
		% of using services	16.00	21.15	32.26	18.75	14.29	31.03	
		% of willingness to vaccinate	74.93	9.51	2.88	1.73	8.36	2.59	
		% of the group	12.52	1.59	0.48	0.29	1.40	0.43	16.71
	**Total**	N	1625	156	31	32	203	29	2076
		% of the entire group	78.28	7.51	1.49	1.54	9.78	1.40	100.00
**Chi^2 Pearson: 13.41181, df=5, p=0.01981, N=2076**									

Table S111: Willingness to vaccinate amongst Filipino students with respect to the fact whether the students were taking any supplements during the pandemic.

Would you like to be vaccinated?			Do you take any supplements during the pandemics?				
			Yes, I continue to take those which I was taken before the pandemics and those which I've started during the pandemics	Yes, I've started to take them during the pandemics	Yes, I continue to take those which I was taking before the pandemics	No	In total
	Yes	N	182	490	526	526	1724
		% of taking suplements	76.15	85.37	78.27	89.76	
		% of willingness to vaccinate	10.56	28.42	30.51	30.51	
		% of the group	8.79	23.66	25.40	25.40	83.24
	No	N	57	84	146	60	347
		% of taking supplements	23.85	14.63	21.73	10.24	
		% of willingness to vaccinate	16.43	24.21	42.07	17.29	
		% of the group	2.75	4.06	7.05	2.90	16.76
	Total	N	239	574	672	586	2071
		% of the entire group	11.54	27.72	32.45	28.30	100.00
Chi² Pearson: 40.22058, df=3, p=0.00000, N=2071							

Table S112: Willingness to vaccinate amongst Filipino students with respect to the fact whether the students are being vaccinated against influenza.

		**Are you being vaccinated against influenza?**			
			**No**	**Yes**	**In total**
**Would you like to be vaccinated?**	**Yes**	N	1128	601	1729
		% of being vaccinated	100.00	63.40	
		% of willingness to vaccinate	65.24	34.76	
		% of the group	54.34	28.95	83.29
	**No**	N	0	347	347
		% of being vaccinated	0.00	36.60	
		% of willingness to vaccinate	0.00	100.00	
		% of the group	0.00	16.71	16.71
	**Total**	N	1128	948	2076
		% of the entire group	54.34	45.66	100.00
**Chi² Pearson: 495.7498, df=1, p=0.0000, N=2076**					

Table S113: Willingness to vaccinate amongst Filipino students with respect to being already vaccinated during the pandemic.

Are you vaccinated against COVID-19?					
			Yes	No	In total
Would you like to be vaccinated?	Yes	N	269	1419	1688
		% of being vaccinated	99.26	78.61	
		% of willingness to vaccinate	15.94	84.06	
	No	N	2	386	388
		% of being vaccinated	0.74	21.39	
		% of willingness to vaccinate	0.52	99.48	
	TOTAL	N	271	1805	2076
Chi² Pearson: 66.0975, df=1, p= 0.00000, N=2076					

Table S114: Willingness to vaccinate amongst Pakistani students with respect to their gender.

Gender					
			**Female**	**Male**	**In total**
**Would you like to be vaccinated?**	**Yes**	N	232	175	407
		% of gender	80.56	80.28	
		% of willingness to vaccinate	57.00	43.00	
		% of the group	45.85	34.58	80.43
	**No**	N	56	43	99

		% of gender	19.44	19.72	
		% of willingness to vaccinate	56.57	43.43	
		% of the group	11.07	8.50	19.57
	**Total**	N	288	218	506
		% of the entire group	56.92	43.08	100.00
**Chi² Pearson: 0.0061958, df=1, p=0.93726, N=506**					

Table S115: Vaccinated Pakistani students during the pandemic with respect to their gender.

**Are you vaccinated against COVID-19?**				
		**Yes**	**No**	**In total**

**Gender**	**Female**	N	183	105	288
		% of being vaccinated	53.67	63.64	
		% of gender	63.54	36.46	
	**Male**	N	158	60	218
		% of being vaccinated	46.33	36.36	
		% of gender	72.48	27.52	
	**TOTAL**	N	341	165	506
**Chi² Pearson: 4.508, df=1, p=0.033, N=506**					

Table S116: Willingness to vaccinate amongst Pakistani students with respect to their field of study during the pandemic.

			Field of study					
			Medical studies	Social sciences	Technical science	Artistic or humanistic studies	Sciences	In total
Would you like to be vaccinated?	Yes	N	217	79	35	28	48	407
		% of field of study	85.10	77.45	79.55	93.33	64.00	
		% of willingness to vaccinate	53.32	19.41	8.60	6.88	11.79	
		% of the group	42.89	15.61	6.92	5.53	9.49	80.43
	No	N	38	23	9	2	27	99
		% of field of study	14.90	22.55	20.45	6.67	36.00	
		% of willingness to vaccinate	38.38	23.23	9.09	2.02	27.27	
		% of the group	7.51	4.55	1.78	0.40	5.34	19.57
	Total	N	255	102	44	30	75	506
		% of the entire group	50.40	20.16	8.70	5.93	14.82	100.00
Chi² Pearson: 20.16679, df=4, p=0.00046, N=506								

Table S117: Vaccinated Pakistani students with respect to their field of study during the pandemic.

Are you vaccinated against COVID-19?					
			**Yes**	**No**	**In total**
**Field of study**	**Medical studies**	N	188	67	255
		% of being vaccinated	55.13	40.61	
		% of field of study	73.73	26.27	
	**Social sciences**	N	71	31	102
		% of being vaccinated	20.82	18.79	
		% of field of study	69.61	30.39	
	**Technical science**	N	30	14	44
		% of being vaccinated	8.80	8.48	
		% of field of study	68.18	31.82	
	**Artistic or humanistic studies**	N	20	10	30
		% of being vaccinated	5.87	6.06	
		% of field of study	66.67	33.33	
	**Sciences**	N	32	43	75
		% of being vaccinated	9.38	26.06	
		% of field of study	42.67	57.33	
	**TOTAL**	N	341	165	506
**Chi² Pearson: 25.766, df=4, p=0.00004, N=506**					

Table S118: Willingness to vaccinate amongst Pakistani students with respect to the current types of classes/studying type at university during the pandemic.

How do you currently study at your university (during this semester)?								
**Would you like to be vaccinated?**			**Completely online**	**More than 50% classes online**	**About 30-50% classes online**	**Up to 30% classes online**	**Completely through direct contact**	**In total**
	**Yes**	N	70	137	109	36	53	405
		% of studying form	73.68	93.84	72.67	80.00	79.10	
		% of willingness to vaccinate	17.28	33.83	26.91	8.89	13.09	
		% of the group	13.92	27.24	21.67	7.16	10.54	80.52
	**No**	N	25	9	41	9	14	98
		% of studying form	26.32	6.16	27.33	20.00	20.90	
		% of willingness to vaccinate	25.51	9.18	41.84	9.18	14.29	
		% of the group	4.97	1.79	8.15	1.79	2.78	19.48
	**Total**	N	95	146	150	45	67	503
		% of the entire group	18.89	29.03	29.82	8.95	13.32	100.00
**Chi² Pearson: 25.32221, df=4, p=0.00004, N=503**								

Table S119: Vaccinated Pakistani students with respect to the current types of classes/studying type at university during the pandemic.

Are you vaccinated against COVID-19?					
			**Yes**	**No**	**In total**
**Way of conducting classes at the university**	**Completely online**	N	62	33	95
		% of being vaccinated	18.24	20.25	
		% of studying form	65.26	34.74	
	**More than 50% classes online**	N	105	41	146
		% of being vaccinated	30.88	25.15	
		% of studying form	71.92	28.08	
	**About 30-50% classes online**	N	92	58	150
		% of being vaccinated	27.06	35.58	
		% of studying form	61.33	38.67	
	**Up to 30% classes online**	N	35	10	45
		% of being vaccinated	10.29	6.13	
		% of studying form	77.78	22.22	
	**Completely through direct contact**	N	46	21	67
		% of being vaccinated	13.53	12.88	
		% of studying form	68.66	31.34	
	**TOTAL**	N	340	163	503
**Chi² Pearson: 6.330, df=4, p=0.175, N= 503**					

Table S120: Willingness to vaccinate amongst Pakistani students with respect to their place of residence during the pandemic.

Place of residence				
			**A city with over 600 thous residents**	**In total**
**Would you like to be vaccinated?**	**Yes**	N	407	407
		% of place of residence	80.43	
		% of willingness to vaccinate	100.00	
		% of the group	80.43	80.43
	**No**	N	99	99
		% of place of residence	19.57	
		% of willingness to vaccinate	100.00	
		% of the group	19.57	19.57
	**Total**	N	506	506
		% of the entire group	100.00	100.00

Chi^2 Pearson: 0.000000, df=0, p=1.0000, N=506

Table S121: Willingness to vaccinate amongst Pakistani students with respect to their marital status during the pandemic.

			Marital status				
			**Single**	**Informal relationship**	**Married**	**Divorcee**	**In total**
**Would you like to be vaccinated?**	**Yes**	N	333	42	31	1	407
		% of marital status	83.67	73.68	70.45	14.29	
		% of willingness to vaccinate	81.82	10.32	7.62	0.25	
		% of the group	65.81	8.30	6.13	0.20	80.43
	**No**	N	65	15	13	6	99
		% of marital status	16.33	26.32	29.55	85.71	

		% of willingness to vaccinate	65.66	15.15	13.13	6.06	
		% of the group	12.85	2.96	2.57	1.19	19.57
	**Total**	N	398	57	44	7	506
		% of the entire group	78.66	11.26	8.70	1.38	100.00
**Chi² Pearson: 26.54307, df=3, p=0.00001, N=506**							

Table S122: Willingness to vaccinate amongst Pakistani students with respect to whether the respondents have children.

**Do you have children?**							
		**No**	**I am pregnant or the**	**One child**	**Two children**	**In total**

				wife/partner is pregnant			
**Would you like to be vaccinated?**	**Yes**	N	393	7	1	6	407
		% of having children	82.05	41.18	25.00	100.00	
		% of willingness to vaccinate	96.56	1.72	0.25	1.47	
		% of the group	77.67	1.38	0.20	1.19	80.43
	**No**	N	86	10	3	0	99
		% of having children	17.95	58.82	75.00	0.00	
		% of willingness to vaccinate	86.87	10.10	3.03	0.00	
		% of the group	17.00	1.98	0.59	0.00	19.57
	**Total**	N	479	17	4	6	506
		% of the entire group	94.66	3.36	0.79	1.19	100.00
**Chi² Pearson: 26.70919, df=3, p=0.00001, N=506**							

Table S123: Willingness to vaccinate amongst Pakistani students taking into account with whom the respondents lived during the pandemic.

			I live with:						
			**Alone**	**Roommates**	**Parents**	**Partner or spouse**	**Partner or spouse and children**	**Children**	**In total**
**Would you like to be vaccinated?**	**Yes**	N	15	32	332	19	3	6	407
		% of living with	78.95	78.05	81.98	90.48	23.08	85.71	
		% of willingness to vaccinate	3.69	7.86	81.57	4.67	0.74	1.47	
		% of the group	2.96	6.32	65.61	3.75	0.59	1.19	80.43
	**No**	N	4	9	73	2	10	1	99
		% of living with	21.05	21.95	18.02	9.52	76.92	14.29	
		% of willingness to vaccinate	4.04	9.09	73.74	2.02	10.10	1.01	
		% of the group	0.79	1.78	14.43	0.40	1.98	0.20	19.57
	**Total**	N	19	41	405	21	13	7	506
		% of the entire group	3.75	8.10	80.04	4.15	2.57	1.38	100.00
**Chi^2 Pearson: 29.43220, df=5, p=0.00002, N=506**									

Table S124: Willingness to vaccinate amongst Pakistani students with respect to one's work during the pandemic.

			No, I do not work	I work mentally	I work physically	I run my own business	In total
			**Are you currently working?**				
**Would you like to be vaccinated?**	**Yes**	N	232	112	19	44	407
		% of currently working	77.85	84.21	82.61	84.62	
		% of willingness to vaccinate	57.00	27.52	4.67	10.81	
		% of the group	45.85	22.13	3.75	8.70	80.43
	**No**	N	66	21	4	8	99
		% of currently working	22.15	15.79	17.39	15.38	
		% of willingness to vaccinate	66.67	21.21	4.04	8.08	
		% of the group	13.04	4.15	0.79	1.58	19.57
	**Total**	N	298	133	23	52	506
		% of the entire group	58.89	26.28	4.55	10.28	100.00

Chi² Pearson: 3.114239, df=3, p=0.37434, N=506

Table S125: Willingness to vaccinate amongst Pakistani students with respect to the fact whether one's used psychological/psychiatric services during the pandemic.

Did you use psychological/psychiatric services during the pandemics?						
**Would you like to be vaccinated?**			**I generally do not use the help of a psychologist/psychiatrist**	**I continue to use the services with the same frequency as before the pandemic**	**I have difficulties with contacting a psychiatrist/psychologist due to the epidemic**	**In total**
	**Yes**	N	348	20	39	407
		% of using services	77.85	100.00	100.00	
		% of willingness to vaccinate	85.50	4.91	9.58	
		% of the group	68.77	3.95	7.71	80.43
	**No**	N	99	0	0	99
		% of using services	22.15	0.00	0.00	
		% of willingness to vaccinate	100.00	0.00	0.00	
		% of the group	19.57	0.00	0.00	19.57

	**Total**	N	447	20	39	506
		% of the entire group	88.34	3.95	7.71	100.00
**Chi² Pearson: 16.24560, df=2, p=0.00030, N=506**						

Table S126: Willingness to vaccinate amongst Pakistani students with respect to the fact whether the students were taking any supplements during the pandemic.

**Do you take any supplements during the pandemics?**						
**Would you like to be vaccinated?**			**Yes, I continue to take those which I was taken before the pandemics and those which I've started during the pandemics**	**Yes, I continue to take those which I was taking before the pandemics**	**No**	**In total**
	**Yes**	N	36	259	112	407
		% of taking supplements	100.00	80.43	75.68	

		% of willingness to vaccinate	8.85	63.64	27.52	
		% of the group	7.11	51.19	22.13	80.43
	**No**	N	0	63	36	99
		% of taking supplements	0.00	19.57	24.32	
		% of willingness to vaccinate	0.00	63.64	36.36	
		% of the group	0.00	12.45	7.11	19.57
	**Total**	N	36	322	148	506
		% of the entire group	7.11	63.64	29.25	100.00
**Chi² Pearson: 10.88678, df=2, p=0.00432, N=506**						

Table S127: Willingness to vaccinate amongst Pakistani students with respect to the fact whether the students are being vaccinated against influenza.

**Are you being vaccinated against influenza?**					
			**No**	**Yes**	**In total**
	**Yes**	N	311	96	407
		% of being vaccinated	85.67	67.13	
		% of willingness to vaccinate	76.41	23.59	

**Would you like to be vaccinated?**		% of the group	61.46	18.97	80.43
	**No**	N	52	47	99
		% of being vaccinated	14.33	32.87	
		% of willingness to vaccinate	52.53	47.47	
		% of the group	10.28	9.29	19.57
	**Total**	N	363	143	506
		% of the entire group	71.74	28.26	100.00
**Chi² Pearson: 22.41195, df=1, p=0.00000, N=506**					

Table S128: Willingness to vaccinate amongst Pakistani students with respect to being already vaccinated during the pandemic.

Are you vaccinated against COVID-19?					
			**Yes**	**No**	**In total**
**Would you like to be vaccinated?**	**Yes**	N	341	66	407
		% of being vaccinated	100.00	40.00	
		% of willingness to vaccinate	83.78	16.22	
	**No**	N	0	99	99
		% of being vaccinated	0.00	60.00	
		% of willingness to vaccinate	0.00	100.00	
	**TOTAL**	N	341	165	506
**Chi² Pearson: 254.367, df=1, p=0.0000, N=506**					

Table S129: Willingness to vaccinate amongst Vietnamese students with respect to their gender.

**Gender**					
			**Female**	**Male**	**In total**
**Would you like to be vaccinated?**	**Yes**	N	54	17	71
		% of gender	72.00	73.91	
		% of willingness to vaccinate	76.06	23.94	
		% of the group	55.10	17.35	72.45
	**No**	N	21	6	27
		% of gender	28.00	26.09	
		% of willingness to vaccinate	77.78	22.22	
		% of the group	21.43	6.12	27.55
	**Total**	N	75	23	98
		% of the entire group	76.53	23.47	100.00
**Chi^2 Pearson: 0.0322733, df=1, p=0.85743, N=98**					

S130: Vaccinated Vietnamese students during the pandemic with respect to their gender.

Are you vaccinated against COVID-19?					
**Gender**			**Yes**	**No**	**In total**
	**Female**	N	7	68	75
		% of being vaccinated	70.00	77.27	
		% of gender	9.33	90.67	
	**Male**	N	3	20	23
		% of being vaccinated	30.00	22.73	
		% of gender	13.04	86.96	
	**TOTAL**	N	10	88	98
**Chi² Pearson: 0.264, df=1, p=0.607, N=98**					

Table S131: Willingness to vaccinate amongst Vietnamese students with respect to their field of study during the pandemic.

Field of study								
			**Medical studies**	**Social sciences**	**Technical science**	**Artistic or humanistic studies**	**Sciences**	**In total**
**Would you like to be vaccinated?**	**Yes**	N	46	18	3	1	3	71
		% of field of study	74.19	66.67	100.00	33.33	100.00	
		% of willingness to vaccinate	64.79	25.35	4.23	1.41	4.23	
		% of the group	46.94	18.37	3.06	1.02	3.06	72.45
	**No**	N	16	9	0	2	0	27
		% of field of the study	25.81	33.33	0.00	66.67	0.00	
		% of willingness to vaccinate	59.26	33.33	0.00	7.41	0.00	
		% of the group	16.33	9.18	0.00	2.04	0.00	27.55
	**Total**	N	62	27	3	3	3	98
		% of the entire group	63.27	27.55	3.06	3.06	3.06	100.00
**Chi² Pearson: 5.128096, df=4, p=0.27440, N=98**								

Table S132: Vaccinated Vietnamese students with respect to their field of study during the pandemic.

Are you vaccinated against COVID-19?					
			**Yes**	**No**	**In total**
**Field of study**	**Medical studies**	N	8	54	62
		% of being vaccinated	80.00	61.36	
		% of field of study	12.90	87.10	
	**Social sciences**	N	1	26	27
		% of being vaccinated	10.00	29.55	
		% of field of study	3.70	96.30	
	**Technical science**	N	0	3	3
		% of being vaccinated	0.00	3.41	
		% of field of study	0.00	100.00	
	**Artistic or humanistic studies**	N	0	3	3
		% of being vaccinated	0.00	3.41	
		% of field of study	0.00	100.00	
	**Sciences**	N	1	2	3
		% of being vaccinated	10.00	2.27	
		% of field of study	33.33	66.67	
	**TOTAL**	N	10	88	98

Chi² Pearson: 4.171, df=4, p=0.383, N=98

Table S133: Willingness to vaccinate amongst Vietnamese students with respect to the current types of classes/studying type at university during the pandemic.

How do you currently study at your university (during this semester)?								
			Completely online	More than 50% classes online	About 30-50% classes online	Up to 30% classes online	Completely through direct contact	In total
Would you like to be vaccinated?	Yes	N	16	24	19	8	4	71
		% of studying form	80.00	63.16	76.00	80.00	80.00	
		% of willingness to vaccinate	22.54	33.80	26.76	11.27	5.63	
		% of the group	16.33	24.49	19.39	8.16	4.08	72.45
	No	N	4	14	6	2	1	27
		% of studying form	20.00	36.84	24.00	20.00	20.00	
		% of willingness to vaccinate	14.81	51.85	22.22	7.41	3.70	
		% of the group	4.08	14.29	6.12	2.04	1.02	27.55

	**Total**	N	20	38	25	10	5	98
		% of the entire group	20.41	38.78	25.51	10.20	5.10	100.00
**Chi² Pearson: 2.801138, df=4, p=0.59164, N=98**								

Table S134: Vaccinated Vietnamese students with respect to the current types of classes/studying type at university during the pandemic.

**Are you vaccinated against COVID-19?**					
**Way of conducting classes at the university**			**Yes**	**No**	**In total**
	**Completely online**	N	3	17	20
		% of being vaccinated	30.00	19.32	
		% of studying form	15.00	85.00	
	**More than 50% classes online**	N	2	36	38
		% of being vaccinated	20.00	40.91	
		% of studying form	5.26	94.74	

	**About 30-50% classes online**	N	5	20	25
		% of being vaccinated	50.00	22.73	
		% of studying form	20.00	80.00	
	**Up to 30% classes online**	N	0	10	10
		% of being vaccinated	0.00	11.36	
		% of studying form	0.00	100.00	
	**Completely through direct contact**	N	0	5	5
		% of being vaccinated	0.00	5.68	
		% of studying form	0.00	100.00	
	**TOTAL**	N	10	88	98
**Chi² Pearson: 5.837, df=4, p=0.211, N= 98**					

Table S135: Willingness to vaccinate amongst Vietnamese students with respect to their place of residence during the pandemic.

**Place of residence**							
		**A city with over 600 thous residents**	**A city between 100 and 600 thous residents**	**A city between 20 and 100**	**The town below 20 thous residents**	**Village**	**In total**

**Would you like to be vaccinated?**					**thous residents**			
	**Yes**	N	55	5	1	1	9	71
		% of palce of residence	68.75	100.00	50.00	100.00	90.00	
		% of willingness to vaccinate	77.46	7.04	1.41	1.41	12.68	
		% of the group	56.12	5.10	1.02	1.02	9.18	72.45
	**No**	N	25	0	1	0	1	27
		% of place of residence	31.25	0.00	50.00	0.00	10.00	
		% of willingness to vaccinate	92.59	0.00	3.70	0.00	3.70	
		% of the group	25.51	0.00	1.02	0.00	1.02	27.55
	**Total**	N	80	5	2	1	10	98
		% of the entire group	81.63	5.10	2.04	1.02	10.20	100.00

Chi^2 Pearson: 4.878273, df=4, p=0.30002, N=98

Table S136: Willingness to vaccinate amongst Vietnamese students with respect to their marital status during the pandemic.

Marital status				
Would you like to be vaccinated?			**Single**	**In total**
	**Yes**	N	71	71
		% of marital status	72.45	
		% of willingness to vaccinate	100.00	
		% of the group	72.45	72.45
	**No**	N	27	27
		% of marital status	27.55	
		% of willingness to vaccinate	100.00	
		% of the group	27.55	27.55
	**Total**	N	98	98
		% of the entire group	100.00	100.00
**Chi² Pearson: 0.000000, df=0, p=1.0000, N=98**				

Table S137: Willingness to vaccinate amongst Vietnamese students with respect to whether the respondents have children.

Do you have children?				
			**No**	**In total**
**Would you like to be vaccinated?**	**Yes**	N	71	71
		% of having children	72.45	
		% of willingness to vaccinate	100.00	
		% of the group	72.45	72.45
	**No**	N	27	27
		% of having children	27.55	
		% of willingness to vaccinate	100.00	
		% of the group	27.55	27.55
	**Total**	N	98	98
		% of the entire group	100.00	100.00
**Chi^2 Pearson: 0.000000, df=0, p=1.0000, N=98**				

Table S137: Willingness to vaccinate amongst Vietnamese students taking into account with whom the respondents lived during the pandemic.

I live with:						
			**Alone**	**Roommates**	**Parents**	**In total**
**Would you like to be vaccinated?**	**Yes**	N	22	25	24	71
		% of living with	88.00	69.44	64.86	
		% of willingness to vaccinate	30.99	35.21	33.80	
		% of the group	22.45	25.51	24.49	72.45
	**No**	N	3	11	13	27
		% of living with	12.00	30.56	35.14	
		% of willingness to vaccinte	11.11	40.74	48.15	
		% of the group	3.06	11.22	13.27	27.55
	**Total**	N	25	36	37	98
		% of the entire group	25.51	36.73	37.76	100.00
**Chi^2 Pearson: 4.257939, df=2, p=0.11896, N=98**						

Table S138: Willingness to vaccinate amongst Vietnamese students with respect to one's work during the pandemic.

			**Are you currently working?**				
			**No, I do not work**	**I work mentally**	**I work physically**	**I run my own business**	**In total**
**Would you like to be vaccinated?**	**Yes**	N	29	37	2	3	71
		% of currently working	74.36	69.81	100.00	75.00	
		% of willingness to vaccinate	40.85	52.11	2.82	4.23	
		% of the group	29.59	37.76	2.04	3.06	72.45
	**No**	N	10	16	0	1	27
		% of currently working	25.64	30.19	0.00	25.00	
		% of willingness to vaccinate	37.04	59.26	0.00	3.70	
		% of the group	10.20	16.33	0.00	1.02	27.55
	**Total**	N	39	53	2	4	98
		% of the entire group	39.80	54.08	2.04	4.08	100.00
**Chi² Pearson: 1.029616, df=3, p=0.79409, N=98**							

Table S139: Willingness to vaccinate amongst Vietnamese students with respect to the fact whether one's used psychological/psychiatric services before the pandemic outbreak.

Did you use psychological/psychiatric services before the pandemics broke out?					
			**No**	**Yes, psychological**	**In total**
**Would you like to be vaccinated?**	**Yes**	N	71	0	71
		% of using services	73.20	0.00	
		% of willingness to vaccinate	100.00	0.00	
		% of the group	72.45	0.00	72.45
	**No**	N	26	1	27
		% of using services	26.80	100.00	

		% of willingness to vaccinate	96.30	3.70	
		% of the group	26.53	1.02	27.55
	**Total**	N	97	1	98
		% of the entire group	98.98	1.02	100.00
**Chi[2] Pearson: 2.656739, df=1, p=0.10311, N=98**					

Table S140: Willingness to vaccinate amongst Vietnamese students with respect to the fact whether one's used psychological/psychiatric services during the pandemic.

**Did you use psychological/psychiatric services during the pandemics?**

Would you like to be vaccinated?			I generally do not use the help of a psychologist/psychiatrist	I had to start using the services of a psychologist/psychiatrist because I feel worse	I continue to use the services with the same frequency as before the pandemic	I need more frequent visits because I feel worse	I have difficulties with contacting a psychiatrist/psychologist due to the epidemic	I go to visits less often due to the improvement of my well-being	In total
	Yes	N	68	1	0	1	0	1	71
		% of using services	73.12	100.00		50.00	0.00	100.00	
		% of willingness to vaccinate	95.77	1.41	0.00	1.41	0.00	1.41	
		% of the group	69.39	1.02	0.00	1.02	0.00	1.02	72.45
	No	N	25	0	0	1	1	0	27
		% of using services	26.88	0.00		50.00	100.00	0.00	
		% of willingness to vaccinate	92.59	0.00	0.00	3.70	3.70	0.00	
		% of the group	25.51	0.00	0.00	1.02	1.02	0.00	27.55
	Total	N	93	1	0	2	1	1	98
		% of the entire group	94.90	1.02	0.00	2.04	1.02	1.02	100.00
Chi^2 Pearson: 3.916020, df=5, p=0.56157, N=98									

Table S141: Willingness to vaccinate amongst Vietnamese students with respect to the fact whether the students were taking any supplements during the pandemic.

Do you take any supplements during the pandemics?							
**Would you like to be vaccinated?**			**Yes, I continue to take those which I was taken before the pandemics and those which I've started during the pandemics**	**Yes, I've started to take them during the pandemics**	**Yes, I continue to take those which I was taking before the pandemics**	**No**	**In total**
	**Yes**	N	3	4	12	52	71
		% of taking supplements	75.00	100.00	63.16	73.24	
		% of willingness to vaccinate	4.23	5.63	16.90	73.24	
		% of the group	3.06	4.08	12.24	53.06	72.45
	**No**	N	1	0	7	19	27
		% of taking supplements	25.00	0.00	36.84	26.76	
		% of willingness to vaccinate	3.70	0.00	25.93	70.37	
		% of the group	1.02	0.00	7.14	19.39	27.55
	**Total**	N	4	4	19	71	98
		% of the entire group	4.08	4.08	19.39	72.45	100.00
**Chi^2 Pearson: 2.378099, df=3, p=0.49772, N=98**							

Table S142: Willingness to vaccinate amongst Vietnamese students with respect to the fact whether the students are being vaccinated against influenza.

Are you being vaccinated against influenza?					
			**No**	**Yes**	**In total**
**Would you like to be vaccinated?**	**Yes**	N	44	27	71
		% of being vaccinated	66.67	84.38	
		% of willingness to vaccinate	61.97	38.03	
		% of the group	44.90	27.55	72.45
	**No**	N	22	5	27
		% of being vaccinated	33.33	15.63	
		% of willingness to vaccinate	81.48	18.52	
		% of the group	22.45	5.10	27.55
	**Total**	N	66	32	98
		% of the entire group	67.35	32.65	100.00
**Chi^2 Pearson: 3.385737, df=1, p=0.06576, N=98**					

Table S143: Willingness to vaccinate amongst Vietnamese students with respect to being already vaccinated during the pandemic.

Are you vaccinated against COVID-19?					
			**Yes**	**No**	**In total**
**Would you like to be vaccinated?**	**Yes**	N	10	61	71
		% of being vaccinated	100.00	69.32	
		% of willingness to vaccinate	14.08	85.92	
	**No**	N	0	27	27
		% of being vaccinated	0.00	30.68	
		% of willingness to vaccinate	0.00	100.00	
	**TOTAL**	N	10	88	98
**Chi² Pearson: 4.2349, df=1, p= 0.039, N=98**					

Table S144: Willingness to vaccinate amongst Chinese students with respect to their gender.

Gender					
			**Female**	**Male**	**In total**
**Would you like to be vaccinated?**	**Yes**	N	294	66	360
		% of gender	72.24	68.75	
		% of willingness to vaccinate	81.67	18.33	
		% of the group	58.45	13.12	71.57
	**No**	N	113	30	143
		% of gender	27.76	31.25	
		% of willingness to	79.02	20.98	

		vaccinate			
		% of the group	22.47	5.96	28.43
	**Total**	N	407	96	503
		% of the entire group	80.91	19.09	100.00
**Chi^2 Pearson: 0.4638932, df=1, p=0.49581, N=503**					

Table S145: Vaccinated Chinese students during the pandemic with respect to their gender.

**Are you vaccinated against COVID-19?**					
			**Yes**	**No**	**In total**
	**Female**	N	6	402	408
		% of being vaccinated	46.15	81.87	

**Gender**		% of gender	1.47	98.53	
	**Male**	N	7	89	96
		% of being vaccinated	53.85	18.13	
		% of gender	7.29	92.71	
	**TOTAL**	N	13	491	504
**Chi² Pearson: 10.479, df=1, p=0.001, N=504**					

Table S146: Willingness to vaccinate amongst Chinese students with respect to their field of study during the pandemic.

<table>
<tr><th colspan="9">Field of study</th></tr>
<tr><th rowspan="13">Would you like to be vaccinated?</th><th colspan="2"></th><th>Medical studies</th><th>Social sciences</th><th>Technical science</th><th>Artistic or humanistic studies</th><th>Sciences</th><th>In total</th></tr>
<tr><th rowspan="4">Yes</th><td>N</td><td>2</td><td>31</td><td>7</td><td>311</td><td>9</td><td>360</td></tr>
<tr><td>% of field of study</td><td>100.00</td><td>73.81</td><td>58.33</td><td>71.82</td><td>64.29</td><td rowspan="2"></td></tr>
<tr><td>% of willingness to vaccinate</td><td>0.56</td><td>8.61</td><td>1.94</td><td>86.39</td><td>2.50</td></tr>
<tr><td>% of the group</td><td>0.40</td><td>6.16</td><td>1.39</td><td>61.83</td><td>1.79</td><td>71.57</td></tr>
<tr><th rowspan="4">No</th><td>N</td><td>0</td><td>11</td><td>5</td><td>122</td><td>5</td><td>143</td></tr>
<tr><td>% of field of study</td><td>0.00</td><td>26.19</td><td>41.67</td><td>28.18</td><td>35.71</td><td rowspan="2"></td></tr>
<tr><td>% of willingness to vaccinate</td><td>0.00</td><td>7.69</td><td>3.50</td><td>85.31</td><td>3.50</td></tr>
<tr><td>% of the group</td><td>0.00</td><td>2.19</td><td>0.99</td><td>24.25</td><td>0.99</td><td>28.43</td></tr>
<tr><th rowspan="2">Total</th><td>N</td><td>2</td><td>42</td><td>12</td><td>433</td><td>14</td><td>503</td></tr>
<tr><td>% of the entire group</td><td>0.40</td><td>8.35</td><td>2.39</td><td>86.08</td><td>2.78</td><td>100.00</td></tr>
<tr><th colspan="9">Chi² Pearson: 2.310198, df=4, p=0.67891, N=503</th></tr>
</table>

Table S147: Vaccinated Chinese students with respect to their field of study during the pandemic.

Are you vaccinated against COVID-19?					
**Field of study**			**Yes**	**No**	**In total**
	**Medical studies**	N	0	2	2
		% of being vaccinated	0.00	0.41	
		% of field of study	0.00	100.00	
	**Social sciences**	N	3	39	42
		% of being vaccinated	23.08	7.94	
		% of field of study	7.14	92.86	
	**Technical science**	N	6	6	12
		% of being vaccinated	46.15	1.22	
		% of field of study	50.00	50.00	
	**Artistic or humanistic studies**	N	4	430	434
		% of being vaccinated	30.77	87.58	
		% of field of study	0.92	99.08	
	**Sciences**	N	0	14	14
		% of being vaccinated	0.00	2.85	
		% of field of study	0.00	100.00	
	**TOTAL**	N	13	491	504
**Chi^2 Pearson: 116.037, df=4, p=0.0000, N=504**					

Table S148: Willingness to vaccinate amongst Chinese students with respect to the current types of classes/studying type at university during the pandemic.

How do you currently study at your university (during this semester)?								
**Would you like to be vaccinated?**			**Completely online**	**More than 50% classes online**	**About 30-50% classes online**	**Up to 30% classes online**	**Completely through direct contact**	**In total**
	**Yes**	N	33	27	20	55	225	360
		% of studying form	70.21	77.14	66.67	78.57	70.09	
		% of willingness to vaccinate	9.17	7.50	5.56	15.28	62.50	
		% of the group	6.56	5.37	3.98	10.93	44.73	71.57
	**No**	N	14	8	10	15	96	143
		% of studying form	29.79	22.86	33.33	21.43	29.91	
		% of willingness to vaccinate	9.79	5.59	6.99	10.49	67.13	
		% of the group	2.78	1.59	1.99	2.98	19.09	28.43
	**Total**	N	47	35	30	70	321	503
		% of the entire group	9.34	6.96	5.96	13.92	63.82	100.00
**Chi² Pearson: 2.961641, df=4, p=0.56427, N=503**								

Table S149: Vaccinated Chinese students with respect to the current types of classes/studying type at university during the pandemic.

Are you vaccinated against COVID-19?					
			**Yes**	**No**	**In total**
**Way of conducting classes at the university**	**Completely online**	N	3	44	47
		% of being vaccinated	23.08	8.96	
		% of studying form	6.38	93.62	
	**More than 50% classes online**	N	2	34	36
		% of being vaccinated	15.38	6.92	
		% of studying form	5.56	94.44	
	**About 30-50% classes online**	N	3	27	30
		% of being vaccinated	23.08	5.50	
		% of studying form	10.00	90.00	
	**Up to 30% classes online**	N	1	69	70
		% of being vaccinated	7.69	14.05	
		% of studying form	1.43	98.57	
	**Completely through direct contact**	N	4	317	321
		% of being vaccinated	30.77	64.56	
		% of studying form	1.25	98.75	
	**TOTAL**	N	13	491	504
**Chi² Pearson: 13.188, df=4, p=0.01, N=504**					

Table S150: Willingness to vaccinate amongst Chinese students with respect to their place of residence during the pandemic.

Place of residence								
			**A city with over 600 thous residents**	**A city between 100 and 600 thous residents**	**A city between 20 and 100 thous residents**	**The town below 20 thous residents**	**Village**	**In total**
**Would you like to be vaccinated?**	**Yes**	N	160	17	36	49	98	360
		% of place of residence	76.56	65.38	61.02	68.06	71.53	
		% of willingness to vaccinate	44.44	4.72	10.00	13.61	27.22	
		% of the group	31.81	3.38	7.16	9.74	19.48	71.57
	**No**	N	49	9	23	23	39	143
		% of place of residence	23.44	34.62	38.98	31.94	28.47	
		% of willingness to vaccinate	34.27	6.29	16.08	16.08	27.27	
		% of the group	9.74	1.79	4.57	4.57	7.75	28.43
	**Total**	N	209	26	59	72	137	503
		% of the entire group	41.55	5.17	11.73	14.31	27.24	100.00
**Chi² Pearson: 6.707889, df=4, p=0.15215, N=503**								

Table S151: Willingness to vaccinate amongst Chinese students with respect to their marital status during the pandemic.

Marital status							
			**Single**	**Informal relationship**	**Married**	**Divorcee**	**In total**
**Would you like to be vaccinated?**	**Yes**	N	297	4	58	1	360
		% of marital status	72.62	80.00	66.67	50.00	
		% of willingness to vaccinate	82.50	1.11	16.11	0.28	
		% of the group	59.05	0.80	11.53	0.20	71.57
	**No**	N	112	1	29	1	143
		% of marital status	27.38	20.00	33.33	50.00	
		% of willingness to vaccinate	78.32	0.70	20.28	0.70	

		% of the group	22.27	0.20	5.77	0.20	28.43
	Total	N	409	5	87	2	503
		% of the entire group	81.31	0.99	17.30	0.40	100.00
**Chi² Pearson: 1.879962, df=3, p=0.59769, N=503**							

Table S152: Willingness to vaccinate amongst Chinese students with respect to whether the respondents have children.

**Do you have children?**								
			**No**	**I am pregnant or the wife/partner is pregnant**	**One child**	**Two children**	**More than two children**	**In total**
	**Yes**	N	354	1	4	1	0	360

**Would you like to be vaccinated?**		% of having children	71.95	50.00	100.00	25.00	0.00	
		% of willingness to vaccinate	98.33	0.28	1.11	0.28	0.00	
		% of the group	70.38	0.20	0.80	0.20	0.00	71.57
	**No**	N	138	1	0	3	1	143
		% of having children	28.05	50.00	0.00	75.00	100.00	
		% of willingness to vaccinate	96.50	0.70	0.00	2.10	0.70	
		% of the group	27.44	0.20	0.00	0.60	0.20	28.43
	**Total**	N	492	2	4	4		503
		% of the entire group	97.81	0.40	0.80	0.80	0.20	100.00
**Chi² Pearson: 8.862400, df=4, p=0.06463, N=503**								

Table S153: Willingness to vaccinate amongst Chinese students taking into account with whom the respondents lived during the pandemic.

I live with:								
			**Alone**	**Roommates**	**Parents**	**Partner or spouse**	**Children**	**In total**
**Would you like to be vaccinated?**	**Yes**	N	5	289	61	2	3	360
		% of living with	55.56	72.07	70.11	100.00	75.00	
		% of willingness to vaccinate	1.39	80.28	16.94	0.56	0.83	
		% of the group	0.99	57.46	12.13	0.40	0.60	71.57
	**No**	N	4	112	26	0	1	143
		% of living with	44.44	27.93	29.89	0.00	25.00	
		% of willingness to vaccinte	2.80	78.32	18.18	0.00	0.70	
		% of the group	0.80	22.27	5.17	0.00	0.20	28.43
	**Total**	N	9	401	87	2	4	503
		% of the entire group	1.79	79.72	17.30	0.40	0.80	100.00
**Chi² Pearson: 2.091761, df=4, p=0.71889, N=503**								

Table S154: Willingness to vaccinate amongst Chinese students with respect to one's work during the pandemic.

Are you currently working?						
			**No, I do not work**	**I work mentally**	**I work physically**	**In total**
**Would you like to be vaccinated?**	**Yes**	N	330	4	26	360
		% of currently working	71.74	80.00	68.42	
		% of willingness to vaccinate	91.67	1.11	7.22	
		% of the group	65.61	0.80	5.17	71.57
	**No**	N	130	1	12	143
		% of currently working	28.26	20.00	31.58	
		% of willingness to vaccinate	90.91	0.70	8.39	
		% of the group	25.84	0.20	2.39	28.43
	**Total**	N	460	5	38	503
		% of the entire group	91.45	0.99	7.55	100.00
**Chi^2 Pearson: 0.366285, df=2, p=0.83265, N=503**						

Table S155: Willingness to vaccinate amongst Chinese students with respect to the fact whether one's used psychological/psychiatric services before the pandemic outbreak.

			Did you use psychological/psychiatric services before the pandemics broke out?				
			**No**	**Yes, psychological**	**Yes, psychiatric**	**Yes, psychological and psychiatric**	**In total**
**Would you like to be vaccinated?**	**Yes**	N	348	8	2	2	360
		% of using services	71.31	72.73	100.00	100.00	
		% of willingness to vaccinate	96.67	2.22	0.56	0.56	
		% of the group	69.18	1.59	0.40	0.40	71.57
	**No**	N	140	3	0	0	143
		% of using services	28.69	27.27	0.00	0.00	
		% of willingness to vaccinate	97.90	2.10	0.00	0.00	
		% of the group	27.83	0.60	0.00	0.00	28.43
	**Total**	N	488	11	2	2	503
		% of the entire group	97.02	2.19	0.40	0.40	100.00
**Chi^2 Pearson: 1.612223, df=3, p=0.65662, N=503**							

Table S156: Willingness to vaccinate amongst Chinese students with respect to the fact whether one's used psychological/psychiatric services during the pandemic.

Did you use psychological/psychiatric services during the pandemics?									
**Would you like to be vaccinated?**			**I generally do not use the help of a psychologist/psychiatrist**	**I had to start using the services of a psychologist/psychiatrist because I feel worse**	**I continue to use the services with the same frequency as before the pandemic**	**I need more frequent visits because I feel worse**	**I have difficulties with contacting a psychiatrist/psychologist due to the epidemic**	**I go to visits less often due to the improvement of my well-being**	**In total**
	**Yes**	N	347	3	4	1	3	2	360
		% of using services	71.40	75.00	66.67	50.00	100.00	100.00	
		% of willingness to vaccinte	96.39	0.83	1.11	0.28	0.83	0.56	
		% of the group	68.99	0.60	0.80	0.20	0.60	0.40	71.57
	**No**	N	139	1	2	1	0	0	143

		% of using services	28.60	25.00	33.33	50.00	0.00	0.00	
		% of willingness to vaccinate	97.20	0.70	1.40	0.70	0.00	0.00	
		% of the group	27.63	0.20	0.40	0.20	0.00	0.00	28.43
	**Total**	N	486	4	6	2	3	2	503
		% of the entire group	96.62	0.80	1.19	0.40	0.60	0.40	100.00
**Chi² Pearson: 2.544515, df=5, p=0.76978, N=503**									

Table S157: Willingness to vaccinate amongst Chinese students with respect to the fact whether the students were taking any supplements during the pandemic.

**Do you take any supplements during the pandemics?**						
**Would you like to be vaccinated?**		**Yes. I continue to take those which I was taken before the pandemics and those**	**Yes. I've started to take them during the pandemics**	**Yes. I continue to take those which I was taking before the pandemics**	**No**	**In total**

			which I’ve started during the pandemics				
	**Yes**	N	9	1	14	336	360
		% of taking supplements	90.00	33.33	77.78	71.19	
		% of willingness to vaccinate	2.50	0.28	3.89	93.33	
		% of the group	1.79	0.20	2.78	66.80	71.57
	**No**	N	1	2	4	136	143
		% of taking supplements	10.00	66.67	22.22	28.81	
		% of willingness to vaccinate	0.70	1.40	2.80	95.10	
		% of the group	0.20	0.40	0.80	27.04	28.43
	**Total**	N	10	3	18	472	503
		% of the entire group	1.99	0.60	3.58	93.84	100.00
**Chi² Pearson: 4.200045, df=3, p=0.24066, N=503**							

Table S158: Willingness to vaccinate amongst Chinese students with respect to the fact whether the students are being vaccinated against influenza.

Are you being vaccinated against influenza?					
			**No**	**Yes**	**In total**
**Would you like to be vaccinated?**	**Yes**	N	8	351	359
		% of being vaccinated	57.14	71.93	
		% of willingness to vaccinate	2.23	97.77	
		% of the group	1.59	69.92	71.51
	**No**	N	6	137	143
		% of being vaccinated	42.86	28.07	
		% of willingness to vaccinate	4.20	95.80	
		% of the group	1.20	27.29	28.49
	**Total**	N	14	488	502
		% of the entire group	2.79	97.21	100.00
**Chi² Pearson: 1.460051, df=1, p=0.22692, N=502**					

Table S159: Willingness to vaccinate amongst Chinese students with respect to being already vaccinated during the pandemic.

Are you vaccinated against COVID-19?					
**Would you like to be vaccinated?**			**Yes**	**No**	**In total**
	**Yes**	N	8	353	361
		% of being vaccinated	61.54	71.89	
		% of willingness to vaccinate	2.22	97.78	
	**No**	N	5	138	143
		% of being vaccinated	38.46	28.11	
		% of willingness to vaccinte	3.50	96.50	
	**TOTAL**	N	13	491	504
**Chi² Pearson: 0.6682, df=1, p=0.413, N=504**					

Table S160: Reasons for students' hesitancy to vaccinate against COVID-19

What stops you from getting vaccinated?	
1.	I am already vaccinated
2.	There is no possibility to be vaccinated right now
3.	I am not at risk
4.	I have good immunity and will not get COVID-19
5.	I prefer other means of protection against infection
6.	The vaccine was developed too quickly, there is too little / no evidence of efficacy and safety
7.	I have concerns about the contents of the vaccine
8.	There are many vaccine companies on the market, and I don't know which to trust
9.	I've already had COVID-19, I don't need to get vaccinated
10.	The diseases I suffer from
11.	I am concerned about the serious side effects of the vaccine
12.	I do not trust pharmaceutical companies and physicians
13.	I have had a bad experience with vaccines in the past, so I don't want to get vaccinated
14.	Vaccines can cause COVID-19 by itself
15.	The COVID-19 pandemic is made up of the rulers/media and does not require vaccination
16.	COVID-19 is the common cold and does not require immunization
17.	I am not at risk
18.	I don't want to get vaccinated for cultural/religious reasons
19.	I do not have sufficient funds/insurance to afford the vaccine
20.	I am concerned about the segregation of society due to vaccinations
21.	I am waiting
22.	I do not receive any vaccines and I won’t do that in this case as well
23.	Other
